# Antitumor Activity of Quinazolinone Alkaloids Inspired by Marine Natural Products

**DOI:** 10.3390/md16080261

**Published:** 2018-07-31

**Authors:** Solida Long, Diana I. S. P. Resende, Anake Kijjoa, Artur M. S. Silva, André Pina, Tamara Fernández-Marcelo, M. Helena Vasconcelos, Emília Sousa, Madalena M. M. Pinto

**Affiliations:** 1 Laboratório de Química Orgânica e Farmacêutica, Departamento de Ciências Químicas, Faculdade de Farmácia, Universidade do Porto, Rua de Jorge Viterbo Ferreira, 228, 4050-313 Porto, Portugal; up201502099@ff.up.pt (S.L.); dresende@ff.up.pt (D.I.S.P.R.); madalena@ff.up.pt (M.M.M.P.); 2 Interdisciplinary Centre of Marine and Environmental Research (CIIMAR), Terminal de Cruzeiros do Porto de Lexões, Av. General Norton de Matos s/n, 4450-208 Matosinhos, Portugal; ankijjoa@icbas.up.pt; 3 ICBAS—Instituto de Ciências Biomédicas Abel Salazar, Universidade do Porto, Rua de Jorge Viterbo Ferreira, 228, 4050-313 Porto, Portugal; 4 Química Orgânica, Produtos Naturais e Agroalimentares (QOPNA), Department of Chemistry, University of Aveiro, 3810-193 Aveiro, Portugal; artur.silva@ua.pt; 5 i3S—Instituto de Investigação e Inovação em Saúde, Universidade do Porto, 4200-135 Porto, Portugal; andre.fig.pina@gmail.com (A.P.); tamarafmarcelo_@hotmail.com (T.F.-M.); hvasconcelos@ipatimup.pt (M.H.V.); 6 Cancer Drug Resistance Group, IPATIMUP—Institute of Molecular Pathology and Immunology of the University of Porto, 4200-135 Porto, Portugal; 7 Department of Biochemistry, FCUP—Faculty of Sciences of the University of Porto, 4169-007 Porto, Portugal; 8 Laboratório de Microbiologia, Departamento de Ciências Biológicas, Faculdade de Farmácia, Universidade do Porto, 4050-313 Porto, Portugal

**Keywords:** antitumor, enantiomers, fiscalins, quinazolinones, synthesis

## Abstract

Many fungal quinazolinone metabolites, which contain the methyl-indole pyrazino [1,2-*b*]quinazoline-3,6-dione core, have been found to possess promising antitumor activity. The purpose of this work was to synthesize the enantiomeric pairs of two members of this quinazolinone family, to explore their potential as antitumor and their ability to revert multidrug resistance. The marine natural product fiscalin B (**4c**), and antienantiomers (**4b**, **5b**, and **5c**) were synthesized via a one-pot approach, while the syn enantiomers (**4a**, **4d**, **5a**, and **5d**) were synthetized by a multi-step procedure. These strategies used anthranilic acid (**i**), chiral *N*-protected α-amino acids (**ii**), and tryptophan methyl esters (**iii**) to form the core ring of pyrazino[2,1-*b*]quinazoline-3,6-dione scaffold. Four enantiomeric pairs, with different enantiomeric purities, were obtained with overall yields ranging from 7 to 40%. Compounds **4a**–**d** and **5a**–**d** were evaluated for their growth inhibitory effect against two tumor cell lines. Differences between enantiomeric pairs were noted and **5a**–**d** displayed GI_50_ values ranging from 31 to 52 μM, which are lower than those of **4a**–**d**. Nevertheless, no effect on P-glycoprotein (P-gp) modulation was observed for all compounds. This study disclosed new data for fiscalin B (**4c**), as well as for its analogues for a future development of novel anticancer drug leads.

## 1. Introduction

**Figure 1:**
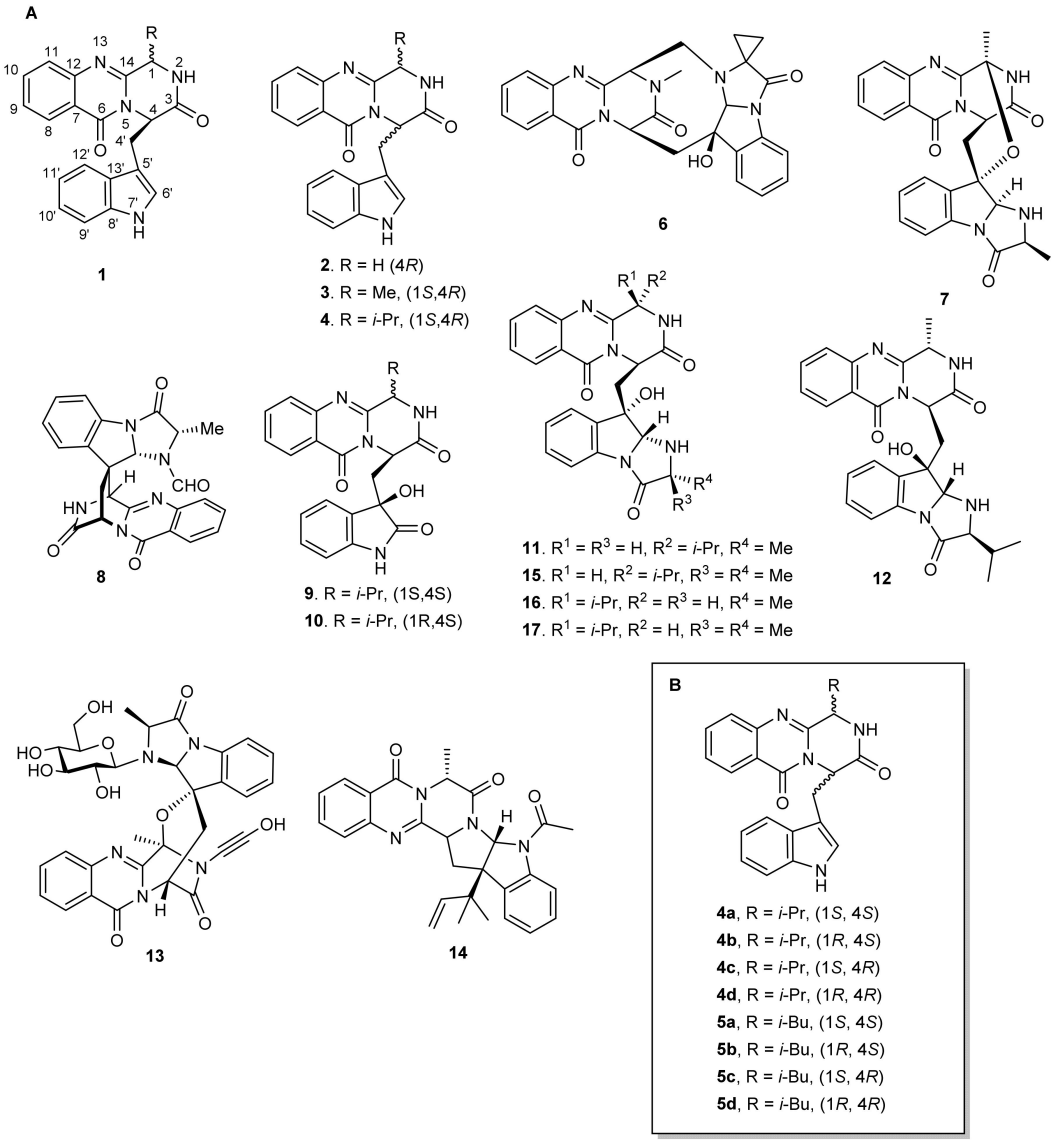
(**A**) Pyrazino[2,1-*b*]quinazoline-3,6-dione ring system (**1**) and some examples of the marine-derived quinazolinone containing alkaloids (**2**–**17**) and (**B**) synthesized diastereomers of fiscalin B and their homologues (**4**–**5**).

During the past ten years, great attention has been focused on drug development from Marine Natural Products (MNPs) as well as on their synthetic and semi-synthetic analogues. While terrestrial sources such as higher plants and microorganisms have reached the limelight, the marine environment increasingly becomes the newest and untapped resource of bioactive compounds [1]. A number of quinazolinone derivatives have played important roles in medicinal chemistry due to their broad spectrum of biological properties such as antibacterial, antifungal, anticonvulsant, anti-inflammatory, anti-HIV, anticancer, and analgesic activities [2,3]. One subclass of quinazolinone-derived Natural Products are the indolylmethyl pyrazinoquinazoline alkaloids (**1**) (Figure 1), characterized by a fused piperazine ring system linked to an indole moiety. So far, approximately 80 secondary metabolites of this subclass, covering structurally diverse compounds, have been isolated from fungi, mainly of marine origin. These include (i) compounds containing only a substituted piperazine ring such as glyantrypine (**2**), isolated from the culture broth of the mangrove-derived fungus *Cladosporium* sp. PJX-41 [4], fumiquinazoline F (**3**), isolated from the marine-derived fungus *Aspergillus fumigatus* strain H1-04 and *Aspergillus* sp. [5,6], and fiscalin B (**4**), isolated from the culture of the fugal strain *Dichotomomyces cejpii* which was recovered from sediments of the Brazilian coast [7]; (ii) compounds with a more complex structure containing several rings such as fumiquinazoline K (**6**), isolated from the Mediterranean sponge-derived fungus *Aspergillus* sp., the soft coral (*Sinularia* sp.)-associated fungus *Aspergillus fumigatus* KMM 4631 [8], and a gorgonian-associated fungus [9]; (iii) *spiro* compounds such as fumiquinazoline C (**7**), isolated from the marine-derived fungus *Aspergillus fumigatus* strain H1-04, and *N*-formyllapatin A (**8**), isolated from the marine-derived fungus *Penicillium adametziodes* (AS-53) [10]; (iv) compounds with complex 3-indolyl groups such as cladoquinazoline (**9**) and epi-cladoquinazoline (**10**), isolated from the mangrove-derived fungus *Cladosporium* sp. PJX-41 [4], neofiscalin A (**11**) from *Neosartorya siamensis* KUFC 6349, which was isolated from a forest soil [11], as well as from *N. siamensis* KUFC 6349, isolated from the sea fan *Rumphella* sp. [12], and fumiquinaziline S (**12**), isolated from a solid-substrate culture of *Aspergillus* sp., collected from a marine-submerged wood [13]; or (v) compounds with indole glucosides such as fumigatoside A (**13**), isolated from *Aspergillus fumigatus* which was derived from the jellyfish *Nemopilema nomurai* [14].

Marine alkaloids containing indolymethyl pyrazinoquinazoline ring system can be considered conformationally constrained peptidomimetics exhibiting very interesting biological properties [15]. For instance, glyantrypine (**2**) is an antibacterial agent, active against *Vibrio harveyi* [16]; fumiquinazolines are antitumor compounds with moderate cytotoxicity [17]; fiscalins are substance P inhibitors and anticancer agents [7,18]; cladoquinazolines (**9** and **10**) are active against influenza A virus (H_1_N_1_); fumiquinazoline S (**12**) exhibits a weak inhibition against Na^+^/K^+^-ATPase, and *N*-acetylardeemin (**14**) is a potent inhibitor of multidrug resistant (MDR) tumor cells [4,13,19,20]. Moreover, the pyrazino[2,1-*b*]quinazoline ring system has been already ascribed as essential for the above-mentioned activities, and its enantioselective effects were observed for their antibacterial activity. For example, neofiscalin A (**11**) showed potent antibacterial activity against Gram-negative bacteria [12,21], whereas fiscalin C (**15**), epi-neofiscalin A (**16**), and epi-fiscalin C (**17**) were inactive in the same study. Interestingly, fiscalin C (**15**) displayed a synergistic effect against methicillin-resistant Staphylococcus aureus (MRSA) when combined with oxacillin [11,22]. Concerning antitumor activity, studies on quinazolinone compounds have mainly focused on natural or synthetic compounds with different substituents at the stereogenic C-1 and C-4; however, there is no report on analogues with different configurations at C-1 and C-4.

Therefore, the aim of this study was to synthesize the diastereomers of fiscalin B (**4c**), i.e., **4a**–**d**, and their homologues **5a**–**d**, to further explore their potential as growth inhibitors of tumor cells, their ability to revert MDR by inhibiting P-gp activity, as well as to perform the SAR study.

## 2. Results and Discussion

### 2.1. Synthesis of Pyrazinoquinazoline Alkaloids

**Scheme 1:**
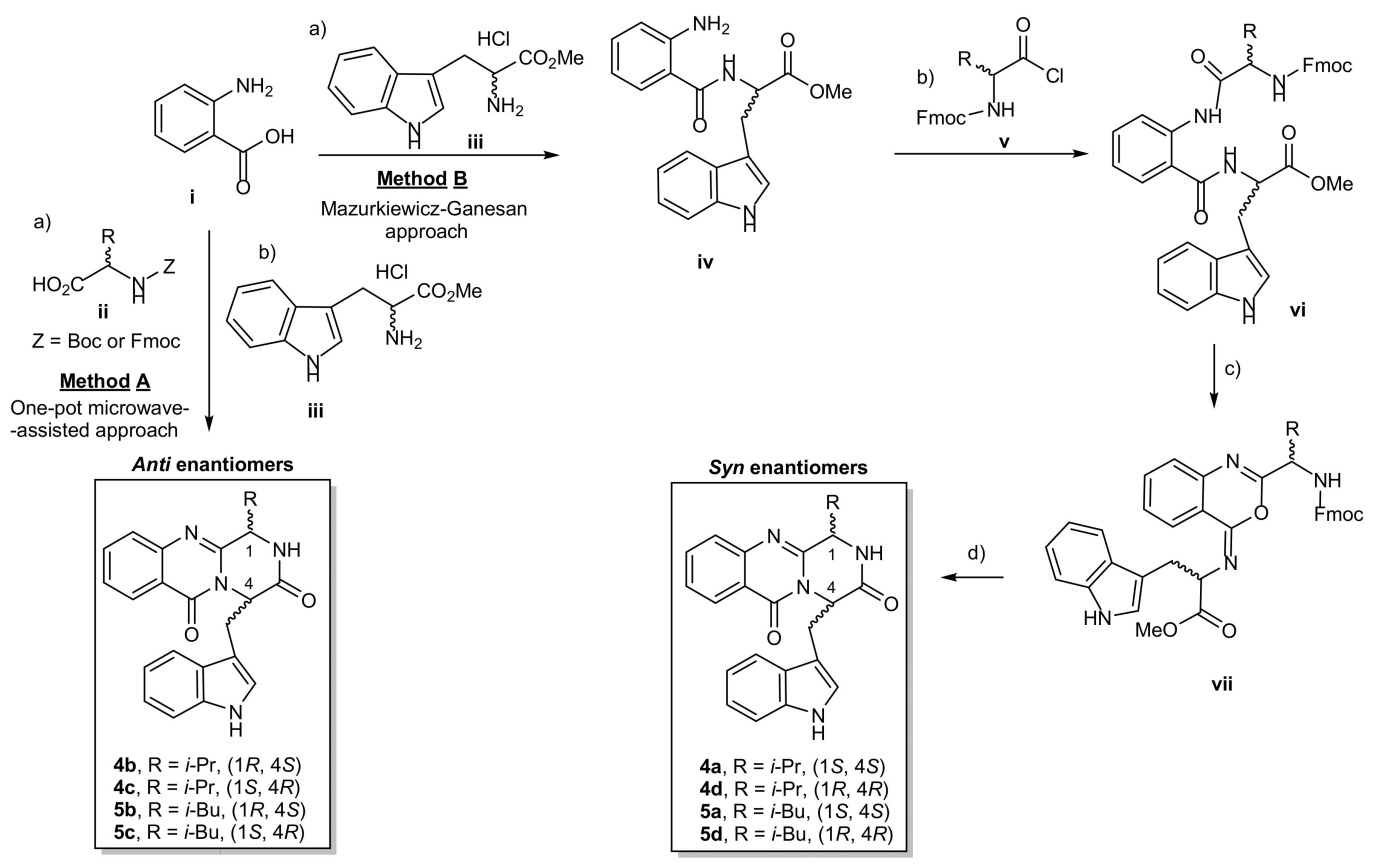
Synthesis of the pyrazinoquinazolinone alkaloids **4** and **5**. (Method A) One-pot microwave-assisted approach. Reagents and conditions: (a) dried-pyridine, (PhO)_3_P, 55 °C, 16–24 h; (b) dried-pyridine, (PhO)_3_P, 220 °C, 1.5 min; (Method B) Mazurkiewicz–Ganesan approach. Reagents and conditions (a) CH_3_CN, TBTU, Et_3_N, rt, 5 h; (b) CH_2_Cl_2_/aq.Na_2_CO_3_, rt, 3 h; (c) dried CH_2_Cl_2_, Ph_3_P, I_2_, EtN(i-Pr)_2_, rt, overnight; (d) piperidine in CH_2_CH_2_, rt, 12 min, then CH_3_CN, DMAP, reflux 19 h. i-Pr = isopropyl; i-Bu = isobutyl, Boc = tert-butyloxycarbonyl; Fmoc = fluorenylmethyloxycarbonyl; DMAP = 4-(dimethylamino) pyridine, TBTU = 1,1,3,3-tetramethylaminium tetrafluoroborate.

The pyrazino[2,1-*b*]quinazoline-3,6-dione ring system (**1**) is the core structure of fumiquinazoline-derived group of alkaloids. There are two main methods to synthesize compounds containing this scaffold (i) the Eguchi-aza Wittig approach that consists of a selective acylation of diketopiperazines with *o*-azidobenzoyl chloride, followed by dehydrative cyclization [23]; and (ii) the Mazurkiewicz–Ganesan approach, consisting of coupling of linear tripeptides followed by the isomerization of 4-imino-4*H*-3,1-benzoxozines to obtain the corresponding quinazolin-4-ones [24,25]. In 2005, Liu et al. [26] reported a highly effective and environmentally friendly approach using a microwave-assisted multicomponent one-pot one step polycondensation of amino acids for the total syntheses of glyantrypine (**2**), fumiquinazoline F (**3**), and fiscalin B (**4**). With this procedure, the authors reported that the addition of a *N*-protected α-amino acid (**ii**) to anthranilic acid (**i**), under a conventional heating condition at 55 °C with triphenylphosphite, (PhO)_3_P, generated the intermediate benzoxazin-4-one, followed by the addition of tryptophan (Trp) ester (**iii**), and then submitted to microwave irradiation at 220 °C for 1.5 min, to furnish the desired final products. Inspired by this simple and highly efficient methodology, we were able to prepare **4** and **5** (Scheme 1, method A). Attempts to obtain the syn enantiomers by this one-pot approach failed since only vestigial amounts could be detected due to the isomerization to the antienantiomers. The antienantiomers **4b**/**4c** and **5b**/**5c** were then obtained, starting from enantiomeric pure amino acids, from which no syn enantiomer were isolated (Entry 1–8, Table 1). Therefore, the syn enantiomers **4a**/**d** and **5a**/**d** were synthesized by the Mazurkiewicz–Ganesan method [25] (Scheme 1, method B) with some modifications. First, coupling of **i** with **iii**, using 1,1,3,3-tetramethylaminium tetrafluoroborate (TBTU) in basic conditions, afforded the dipeptide (**iv**). Coupling of **iv** with *N*-protected α-amino acids chloride (**v**) [27] in a two-phase Schotten–Baumann condition yielded the tripeptides **vi**. The intermediates **vii** were obtained by adding the dehydrating agent, triphenylphosphine (Ph_3_P), to convert β-keto amides (**vi**) to the oxazoles (**vii**), followed by Fmoc-deprotection by 20% piperidine to afford **4a**/**d** and **5a**/**d** with a moderate yields of 21–40%. After purification by chromatographic techniques, the purity of **4a**–**d** and **5a**–**d**, as determined by a reversed-phase HPLC (C18, MeOH:H_2_O; 60:40 or CH_3_CN:H_2_O; 50:50) was found to be higher than 90% (Supplementary Materials, Figures S1–S8). The enantiomers ratio was determined by the chiral HPLC equipped with amylose *tris*-3,5-dimethylphenylcarbamate column, using hexane: ethanol (80:20) as a mobile phase (Table 1, Supplementary Materials, Figures S9–S16).

The overall yield of this one-step method ranged from 7 to 14% with different enantiomeric ratios (Table 1). The low yields of this one-pot reaction were attributed to a high temperature applied in microwave irradiation to convert the intermediate Boc-protected-benzoxazin-4-one to the final products. Moreover, the steric hindrance at C-1 could also be a reason, as previously noted by Liu et al. [26] and Wang et al. [24] in the synthesis of similar compounds. Although a partial epimerization was observed under these conditions, this one-pot procedure was proved successful in providing the pyrazinoquinazolinone scaffold and a series of compounds for further biological investigations. On the other hand, the moderate yields of compounds obtained by Mazurkiewicz–Ganesan method were related to the mild conditions and a multistep approach (Table 1 entry 9–12). In this study, the coupling agent 1-ethyl-3-(3-dimethylaminopropyl)carbodiimide (ECD) was replaced by TBTU, which gave a similar yield for the dipeptide **iv** (81–94%) when compared to the previous reports. The tripeptides **vi** were also obtained in a very high yield (84–94%). The bottleneck of this multistep approach was the conversion of the intermediates **vii** to the final products since their decomposition to form the precursors **vi**, similar to what has been previously report by Ganesan et al. [25], was observed. Compounds **4a**/**d** and **5a**/**d** were purified by preparative TLC (EtOAc:CH_2_Cl_2_:MeOH: 50:47.5:2.5) after refluxing in CH_3_CN in the presence of 4-(dimethylamino)pyridine. The degrees of epimerization of **4a**/**d** and **5a**/**d** by Mazurkiewicz–Ganesan method were found to be lower than those obtained by the microwave method (Table 1). These results could be also associated with the mild conditions used in the multistep approach.

### 2.2. Structure Elucidation

**Figure 2:**
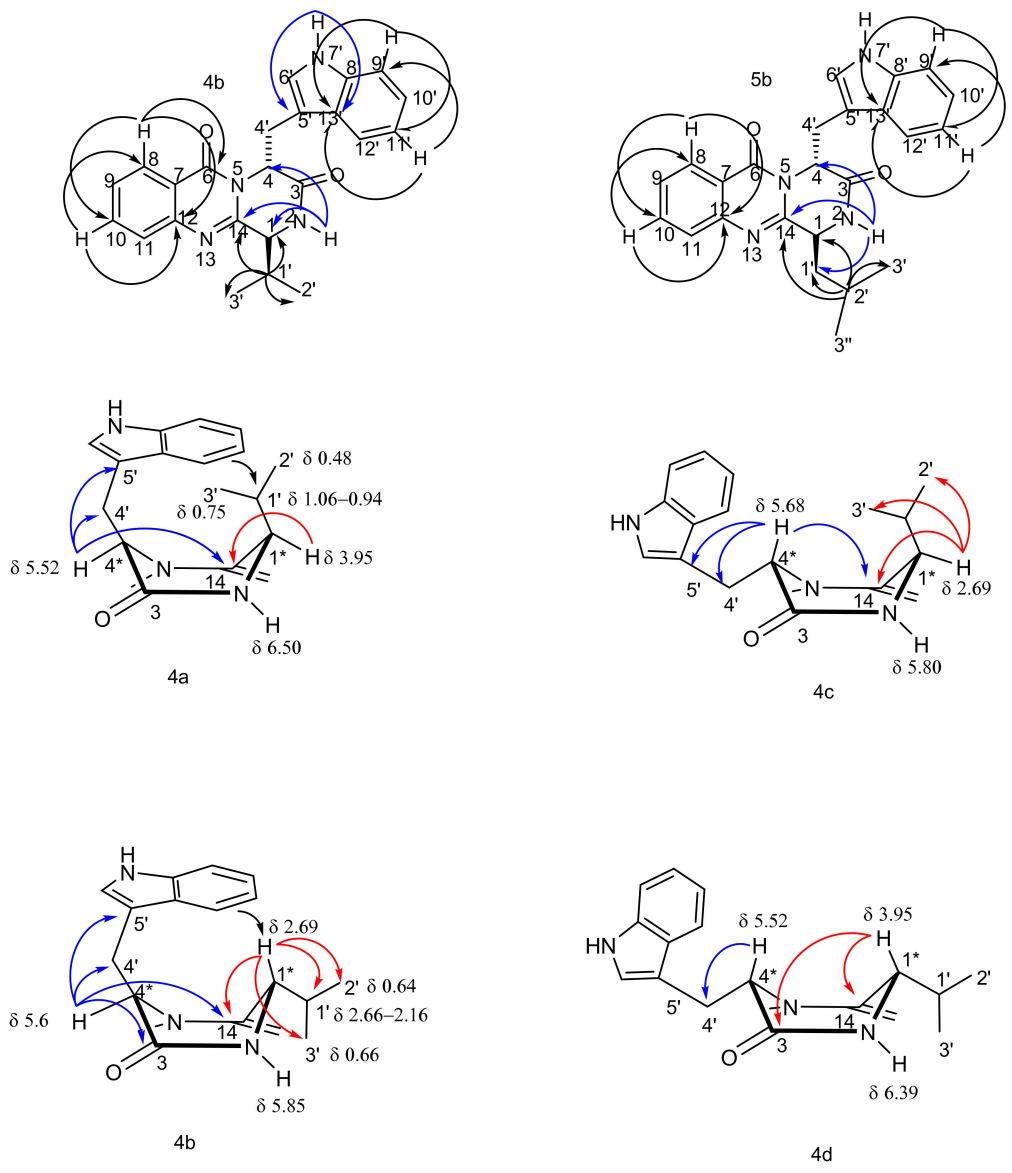
Most relevant chemical shifts and key HMBC correlations of the protons to the stereogenic centers on the piperazine ring of **4a**–**d**.

The structures of the new compounds, **4a**, **4b**, and **4d**, and of the synthetic fiscalin B (**4c**) and their homologues **5a**, **5b**, **5d**, and **5c**, were established by extensive analyses of 1D and 2D NMR spectra and high-resolution mass spectrometry. The ^1^H (Table 2, Supplementary Materials, Figures S17–S30) and ^13^C NMR data (Table 3, Supplementary Materials, Figures S31–S44) of **4c** were in agreement with those reported in the literature for fiscalin B, which was obtained from the marine sources [24,25]. The chemical shift values of some key protons allowed the determination of the relative configurations of both stereogenic centres as well as the conformation of the piperazine ring in **4** and **5**, as shown in **4a**, **4b** and **4c**, **4d** (Figure 2). The substituent on C-4 is always in a pseudoaxial position while H-4 is shifted to δ_H_ 5.52–5.68 in all compounds, showing the characteristic anisotropic effect of the coplanar carbonyl group at C-14 on the quasi-equatorial proton, as explained by Hernández et al. [28]. The difference between anti and syn enantiomers were observed on H-1. In the antienantiomers, the chemical shift value of the axial H-1 of **4b**/**c** and **5b**/**c** at δ_H_ 2.69–2.73 indicates the folding of the C-4-indolyl substituent over the piperazine ring and the isopropyl group. This phenomenon was also reported by Hernández and coworkers for H-1 signals of quinazolinones whose chemical shift values were ca. 3 ppm for the boat conformation and the antienantiomers, due to the absence of the shielding effect by the aromatic ring [28]. Meanwhile, for the syn enantiomers, **4a**/**d** and **5a**/**d**, H-1 chemical shift values were at ca. δ_H_ 3.95–4.32, indicating the shielding effect from the aromatic ring over H-1. The chemical shifts of H-1′ of the isopropyl group were also different between the anti- and the syn enantiomers, being ca. δ_H_ 0.94–1.06 for **4a**/**d** and δ_H_ 2.61–2.66 for **4c**/**d**, indicating the different shielding effect of the aromatic ring on the group on C-1.

HMBC correlations were also used to distinguish the anti-isomers from the *syn* counterparts. For the *anti* isomer **4b** (whose indole moiety derived from l-Trp), H-4 exhibited correlations to C-14, C-5′, C-4′, and C-3 whereas H-1 showed correlations to C-14, C-2′, C-3′, and C-1′. On the contrary, in **4c** (whose indole moiety is derived from d-Trp), the HMBC correlations from H-4 to C-14, C-5′, C-4′ and from H-1 to C-14, C-3′, and C-2′, were observed. For the *syn* isomer **4a** (whose indole moiety derived from l-Trp), the HMBC correlations from H-1 to C-14, and from H-4 to C-14, C-4′, and C-5′ were observed while the HMBC correlations from H-1 to C-3 and C-14 and from H-4 to only C-4′ were observed in the *syn* isomer **4d**. Moreover, the NOESY spectrum revealed the cross peak between the C-1′ methyl groups and H-4 for the *anti* isomer **4c**, while for the *syn* isomer **4a** that correlation was absent (Supplementary Materials, Figures S45–S46). These observations support the identity/identification of the *syn* and *anti* conformational isomers.

### 2.3. Tumor Cell Growth Inhibitory Activity

Compounds **4a**–**d** and **5a**–**d** were tested for their tumor cell growth inhibitory activity against two human tumor cell lines: NCI-H460 (non-small cell lung cancer) and HCT-15 (colorectal adenocarcinoma), using the sulforhodamine B (SRB) colorimetric assay [29]. Five serial dilutions of each compound (at a maximum concentration of 150 μM) were tested for 48 h. Doxorubicin was used as a positive control, and the antitumor activity was reported as GI_50_ (drug concentration that inhibits the growth of cancer cells by 50%).

Compounds **4a**–**d**, **5a**, **5b**, and **5d** were also investigated for their possible modulatory activity of P-gp, a drug efflux pump associated with drug resistance. P-gp activity was determined by an assay which measures the mean fluorescence intensity of cells treated concomitantly with rhodamine 123 (Rh123, a substrate of P-gp), and the tested compounds [20]. The P-gp inhibitory activity of the compounds was tested on a drug resistant cell line which overexpresses P-gp (K562Dox), by measuring the intracellular accumulation of Rh123. After an incubation with the compounds and Rh123, cells were washed, and the fluorescence of Rh123 was detected by flow cytometry in the FL1 channel. The drug sensitive counterpart cells (K562) were used as control. The Rh123 accumulation ratio was calculated as: (Mean FL1_K562Dox+Compound_ − Mean FL1_K562Dox_)/Mean FL1_K562Dox_ [30].

**Figure 3:**
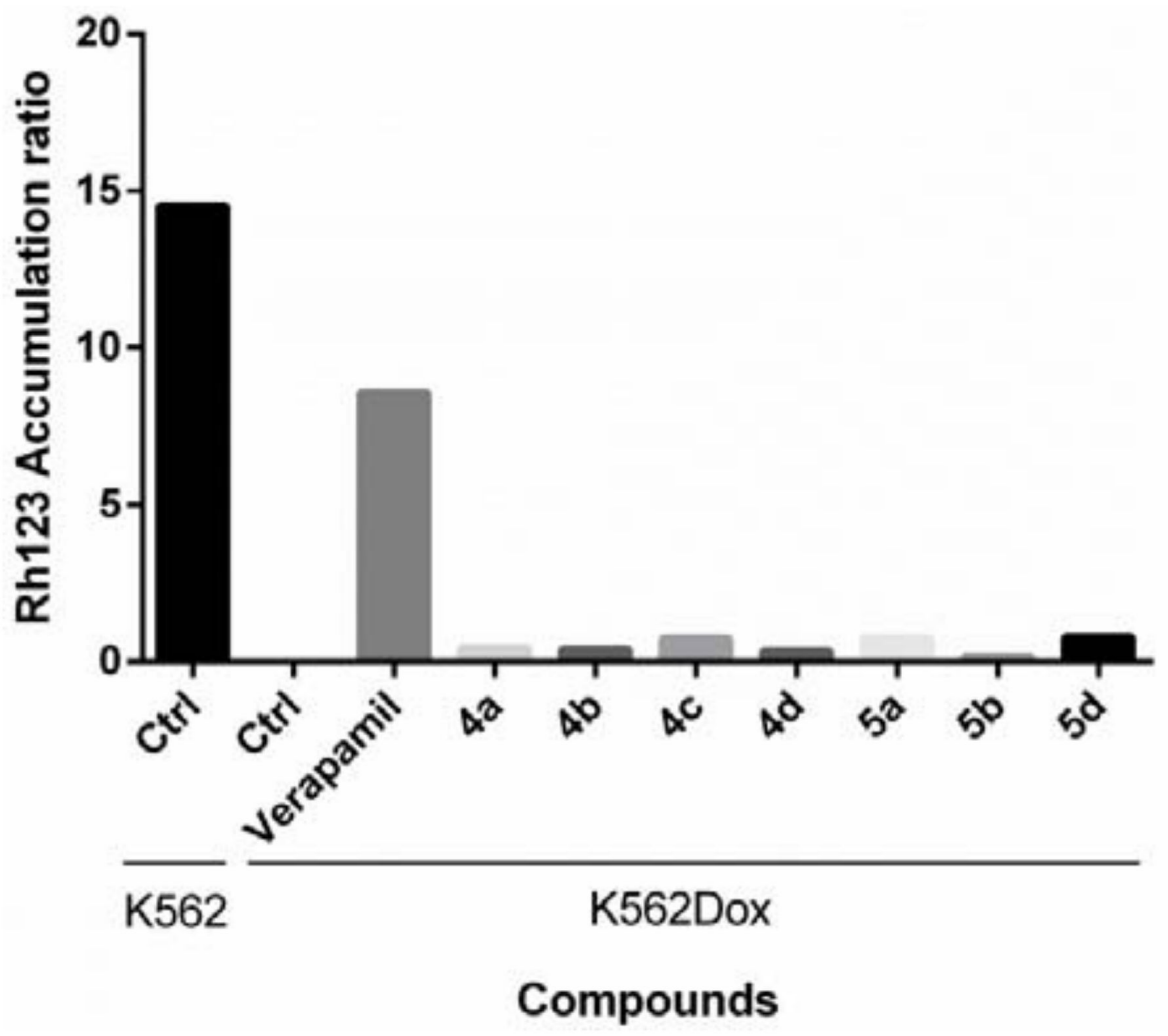
Accumulation ratio of Rh123 in K562 and K562Dox cell lines. Cells were incubated for 1 h with **4a**–**d**, **5a**, **5b**, and **5d** at a final concentration of 10 µM. The activity of **5c** was not analyzed due to its quantity we have obtained. Verapamil (10 µM) was used as a positive control (known P-gp inhibitor), and K562 cells were used as a negative control. The accumulation ratio in the untreated K562Dox cells was defined as zero; any value higher than that represents a potential inhibition of P-gp. Results are the mean of two independent experiments.

All the compounds tested showed weak to moderate activity, with the GI_50_ values ranging from 30 to 80 µM. Some differences were observed among the groups of **4** and **5**. Compounds **4** were more potent in the HCT-15 cell line but exhibited higher GI_50_ values in the NCI-H460 cell lines. On the other hand, members of **5** were more potent than those of **4** in the NCI-H460 cell line. Compound **5c** was the most promising in this panel of cell lines (Table 4). The substituent at C-1 was found to influence the inhibitory effects observed in the NCI-H460 cell. For example, **4a**–**d**, whose C-1 bears the isopropyl group, exhibited GI_50_ values ranging from 57 to 81 µM, while **5a**–**d**, whose C-1 bears the isobutyl group, displayed GI_50_ values ranging from 31 to 42 µM. These findings are in accordance with the SAR obtained with the natural compounds in vitro antitumor assays. For instance, glyantrypine (**2**), whose C-1 bears a hydrogen atom, showed no antitumor activity (GI_50_ > 100 µM) while the analogue, with the phenyl group on C-1, was more active (GI_50_ = 15 µM) [25]. Moreover, fumiquinazolines F (**3**) and G (**4**), whose C-1 bears a methyl group, showed moderate activity against P-388 cells (GI_50_ = 13.5 µM) [31]. Likewise, differences in the inhibitory effects against the two cell lines were observed between enantiomeric pairs; i.e., **4a** (1*S*,4*S*)/**4d** (1*R*,4*R*) and **4b** (1*S*,1*R*)/**4c** (1*R*,4*S*). Significant differences were detected for GI_50_ concentration values in the pair **4a**/**4d** in NCI-H460 cells (*p* = 0.026). Among the fiscalin series such as epi-fiscalin A (**16**, 1*S*,4*S*), epi-fiscalin C (**17**, 1*S*,4*S*), fiscalin F (1*S*,4*S*), and fiscalin C (**15**, 1*R*,4*S*), the configurations of the stereogenic carbons of the isopropyl pyrazinone and imidazolone moieties have already been found to influence the antitumor activity [21,32]. Although this study brought insights into the antitumor activity of fiscalin B (**4c**) and the synthetic analogues, none of the compounds showed any effect on the intracellular accumulation of Rh123, when tested at 10 µM concentrations using verapamil as a positive control for P-gp inhibition (Figure 3).

## 3. Materials and Methods

### 3.1. General Procedure

All reagents were from analytical grade. Dried pyridine and triphenylphosphite were purchased from Sigma (Sigma-Aldrich Co. Ltd., Gillinghan, UK). Protected amino acids (**ii**) and anthranilic acid (**i**) were purchased from TCI (Tokyo Chemical Industry Co. Ltd., Chuo-ku, Tokyo, Japan). Column chromatography purifications were performed using flash silica Merck 60, 230–400 mesh (EMD Millipore corporation, Billerica, MA, USA) and preparative TLC was carried out on precoated plates Merck Kieselgel 60 F_254_ (EMD Millipore corporation, Billerica, MA, USA), spots were visualized with UV light (Vilber Lourmat, Marne-la-Vallée, France). Melting points were measured in a Köfler microscope and are uncorrected. Infrared spectra were recorded in a KBr microplate in a FTIR spectrometer Nicolet iS10 from Thermo Scientific (Waltham, MA, USA) with Smart OMNI-Transmission accessory (Software 188 OMNIC 8.3). ^1^H and ^13^C NMR spectra were recorded in CDCl_3_ (Deutero GmbH, Kastellaun, Germany) at room temperature unless otherwise mentioned on Bruker AMC instrument (Bruker Biosciences Corporation, Billerica, MA, USA), operating at 300 MHz for ^1^H and 75 MHz for ^13^C). Carbons were assigned according to HSQC and or HMBC experiments. Optical rotation was measured at 25 °C using the ADP 410 polarimeter (Bellingham + Stanley Ltd., Tunbridge Wells, Kent, UK), using the emission wavelength of sodium lamp, concentrations are given in g/100 mL. Qualitative GC-MS analyses were performed on a Trace GC 2000 Series ThermoQuest gas chromatography (Thermo Fisher Scientific Inc., Austin, TX, USA) equipped with ion-trap GCQ Plus ThermoQuest Finnigan mass detector (Thermo Fisher Scientific Inc., Austin, TX, USA). Chromatographic separation was achieved using a capillary column (30 m × 0.25 mm × 0.25 μm, cross-linked 5% diphenyl and 95% dimethyl polysiloxane) from Thermo Scientific^TM^ (Thermo Fisher Scientific Inc., Austin, TX, USA) and high-purity helium C-60 as carrier gas. High resolution mass spectra (HRMS) were measured on a Bruker FTMS APEX III mass spectrometer (Bruker Corporation, Billerica, MA, USA) recorded as ESI (Electrospray) made in Centro de Apoio Cientifico e Tecnolόxico á Investigation (CACTI, University of Vigo, Pontevendra, Spain). The purity of synthesized compounds was determined by reversed-phase HPLC with diode array detector (DAD) using C18 column (Kimetex^®^, 2.6 EV0 C18 100 Å, 150 × 4.6 mm), and the mobile phase was methanol: water (60:40) or acetonitrile:water (50:50). Enantiomeric ratio was determined by chiral HPLC (LCMS-2010EV, Shimadzu, Lisbon, Portugal), employing a system equipped with a chiral column (Lux^®^ 5 µm Amylose-1, 250 × 4.6 mm) and UV-detection at 254 nm, mobile phase was hexane:ethanol (80:20) and the flow rate was 0.5 mL/min.

### 3.2. General Conditions for the Synthesis of 4-(1H-Indol-3-ylmethyl)-1-isopropyl-2H-pyrazino[2,1-b]quinazoline-3,6-(1H,4H)-diones (***4b***/***4c***)

In a closed vial, anthranilic acid (**i**) (28 mg, 200 µmol), *N*-Boc-l-valine (**iia**) for **4c** or *N*-Boc-d-valine (**iib**) for **4b** (44 mg, 200 µmol), and triphenylphosphite (63 µL, 220 µmol) were added along with 1 mL of dried pyridine. The vial was heated in heating block with stirring at 55 °C for 16–24 h. After cooling the mixture to room temperature, l-tryptophan methyl ester hydrochloride (**iiia**) for **4b** or d-tryptophan methyl ester hydrochloride (**iiib**) for **4c** (51 mg, 200 µmol) was added, and the mixture was irradiated in the microwave at a constant temperature at 220 °C for 1.5 min. Four reaction mixtures were prepared in the same conditions and treated in parallel. After removing the solvent with toluene, the crude product was purified by flash column chromatography using hexane: EtOAc (60:40) as a mobile phase. The preparative TLC was performed using CH_2_Cl_2_:Me_2_CO (95:5) as mobile phase. The major compound appeared as a black spot with no fluorescence under the UV light. The desired compounds **4b**/**c** were collected as yellow solids. Before analysis, compounds were recrystallized from methanol.

#### 3.2.1. (1*R*,4*S*)-4-(1*H*-Indol-3-ylmethyl)-1-isopropyl-2*H*-pyrazino[2,1-*b*]quinazoline-3,6-(1*H*,4*H*)-dione (**4b**)

Yield: 14%; m.p.: 168–169 °C; [α]_D_ = +65.21 (*c* 0.46; CHCl_3_); IR *v_max_* (KBr) 3411, 3066, 1684, 1596, 1471, 1389, 1293 cm^−1^; ^1^H NMR see Table 2; ^13^C NMR see Table 3; *m*/*z* (rel. intensity, %): 385.9 (M^+^, 2), 257.1 (29), 214.1 (3), 202.0 (7), 171.1 (10), 143.1 (4), 130.1 (100), 103.0 (18), 77.0 (16); (+)-HRESIMS *m*/*z* 387.1810 (M + H)^+^ (calculated for C_23_H_22_N_4_O_2_, 387.1776). 

#### 3.2.2. (1*S*,4*R*)-4-(1*H*-Indol-3-ylmethyl)-1-isopropyl-2*H*-pyrazino[2,1-*b*]quinazoline-3,6-(1*H*,4*H*)-dione (**4c**)

Yield: 8%; m.p.: 168–169 °C; [α]_D_ = −248.1 (*c* 0.43; CHCl_3_); IR *v_max_* (KBr) 3346, 3066, 1683, 1596, 1471, 1389, 1293 cm^−1^; ^1^H NMR see Table 2; ^13^C NMR see Table 3; *m*/*z* (rel. intensity, %): 385.9 (M^+^, 5), 257.1 (32), 298.9 (37), 284.2 (6), 254.0 (15), 238.8 (12), 201.8 (18), 189.0 (35), 171.1 (25), 149.1 (32), 130.1 (100), 103.0 (9), 77.0 (13); (+)-HRESIMS *m*/*z* 387.1809 (M + H)^+^ (calculated for C_23_H_22_N_4_O_2_, 387.1776).

### 3.3. General Condition for the Synthesis of 4-(1H-Indol-3-ylmethyl)-1-isobutyl-2H-pyrazino[2,1-b]quinazoline-3,6-(1H,4H)-diones (***5b***/***5c***)

In a closed vial, anthranilic acid (i) (28 mg, 200 µmol), *N*-Fmoc-l-leucine (**iic**) for **5c** or *N*-Fmoc-d-leucine (**iid**) for **5b** (70.68 mg, 200 µmol), and triphenylphosphite (63 µL, 220 µmol) were added along with 1 mL of dried pyridine. The vial was heated in heating block at 55 °C for 16–24 h. After cooling the mixture to room temperature, we added l-tryptophan methyl ester hydrochloride (**iiia**) for **5b** (51 mg, 200 µmol), or d-tryptophan methyl ester hydrochloride (**iiib**) **5d** (51 mg, 200 µmol), and the mixture was irradiated in the microwave at a constant temperature at 220 °C for 1.5 min. Four reaction mixtures were prepared in the same conditions and treated in parallel. After removing the solvent with toluene, the mixture was purified by flash column chromatography using hexane: EtOAc (60:40) as a mobile phase. The preparative TLC was performed using CH_2_Cl_2_:Me_2_CO (95:5) as a mobile phase. The major compound appeared as a black spot with no fluorescence under UV light. The desired compounds, **5b**/**5c**, were collected as yellow solids. Before analysis, compounds were recrystallized from methanol.

#### 3.3.1. (1*R*,4*S*)-4-(1*H*-Indol-3-ylmethyl)-1-isobutyl-2*H*-pyrazino[2,1-*b*]quinazoline-3,6-(1*H*,4*H*)-dione (**5b**)

Yield: 10%; m.p.: 220 °C; [α]_D_ = +89.74 (*c* 0.52; CHCl_3_); IR *v_max_* (KBr) 3334, 3060, 1686, 1457, 1386, 1291 cm^−1^; ^1^H NMR see Table 2; ^13^C NMR see Table 3; (+)-HRESIMS *m*/*z* 401.1967 (M + H)^+^ (calculated for C_24_H_24_N_4_O_2_, 401.1933).

#### 3.3.2. (1*S*,4*R*)-4-(1*H*-Indol-3-ylmethyl)-1-isobutyl-2*H*-pyrazino[2,1-*b*]quinazoline-3,6-(1*H*,4*H*)-dione (**5c**)

Yield: 8%; m.p.: 219 °C; [α]_D_ = −61.72 (*c* 0.54; CHCl_3_); IR *v_max_* (KBr) 3334, 3060, 1682, 1587, 1396, 1298 cm^−1^; ^1^H NMR see Table 2; ^13^C NMR see Table 3; (+)-HRESIMS *m*/*z* 401.1966 (M + H)^+^ (calculated for C_24_H_24_N_4_O_2_, 401.1933).

### 3.4. General Condition for the Synthesis of Compounds ***4a*** and ***5a***

#### 3.4.1. Synthesis of *N*-(2-Aminobenzoyl)-l-tryptophan methyl ester (**iv-a**)

To a mixture of anthranilic acid (287 mg, 2.39 mmol) and TBTU (920 mg, 2.86 mmol, 1.2 equiv) in acetonitrile (20 mL) was added Et_3_N (833 µL, 4.78 mmol, 2 equiv) and l-tryptophan methyl ester (521 mg, 2.39 mmol) at room temperature. After stirring for 5 h, the reaction mixture was concentrated under reduced pressure. The residue was dissolved in CH_2_Cl_2_ and washed with 1 M HCl, extracted with CH_2_Cl_2_ (3 × 100 mL), dried with Na_2_SO_4_, filtered, and concentrated. The residue was purified by flash chromatography (eluent 1% MeOH in CH_2_Cl_2_) to yield **iv-a** as a white solid (675.5 mg, 96%), m.p. 134–135 °C, IR *v_max_* (KBr): 3424, 1746, 1727, 1645, 1611, 1581; ^1^H NMR (300, MHz, CDCl_3_): 8.15 (s, 1H), 7.56 (d, 1H, *J* = 7.9 Hz), 7.35 (d, 1H, *J* = 8.1 Hz), 7.19–7.15 (m, 3H), 7.09 (t, 1H, *J* = 7.4 Hz), 7.01 (d, 1H, *J* = 2.3 Hz), 6.65 (dd, 1H, *J* = 8,7 and 1.1 Hz), 6.56 (ddd, 1H, *J* = 7.5 and 0.8 Hz), 5.08 (dt, 1H, *J* = 7.5 and 5.3 Hz), 3.71 (s, 3H), 3.43 (m, 2H); ^13^C NMR (75, MHz, CDCl_3_) 172.6 (CO), 168.8 (CO), 148.8 (C), 136.1 (C), 132.6 (CH), 127.6 (CH), 127.6 (C), 122.8 (CH), 122.3 (CH), 119.8 (CH), 118.7 (CH), 117.3 (CH), 116.7 (CH), 115.3 (C), 111.3 (CH), 110.1 (C), 53.1 (CH), 52.7 (CH_3_), 27.7 (CH_2_).

#### 3.4.2. Synthesis of *N*-[9*H*-Fluoren-9-ylmethoxy)carbonyl]-l-valinyl-2-aminobenzoyl-l-tryptophan methyl ester (**vi**-**a**)

To a solution of **iv-a** (160 mg, 0.474 mmol) in dried CH_2_Cl_2_ (10 mL) was added *N*-Fmoc-l-valine-Cl [27] (**v-a**,182 mg, 0.5 mmol). The mixture was stirred for 30 min, followed by addition of aqueous Na_2_CO_3_ (1 M, 8 mL, 8 mmol). After continuous stirring for 3 h, the mixture was extracted with CH_2_Cl_2_ (4 × 100 mL), dried with Na_2_SO_4_, filtered, and concentrated. The residue was purified by flash chromatography (eluent: 5% MeOH in CH_2_Cl_2_ to give **vi-a** as a white solid (296.4 mg, 95%), m.p.: 194.4–196.3 °C, ${\left[\alpha \right]}_{D}^{30}$ = +13.22 (*c* 0.126, CHCl_3_), IR *v_max_* (KBr) 3318, 1727, 1698, 1588 cm^−1^; ^1^H NMR (300, MHz, CDCl_3_): 11.4 (s, 1H), 8.50 (d, 1H, *J* = 8.3 Hz), 8.24 (s, 1H), 7.67 (d, 2H, *J* = 7.4 Hz), 7.57 (d, 1H, *J* = 7.4 Hz), 7.51 (d, *J* = 7.3), 7.43–7.19 (m, 8H), 7.08 (t, 1H, *J* = 7.4 Hz), 6.97 (t, 1H, *J* = 7.4 Hz), 6.91 (d, 1H, *J* = 7.5 Hz) 6.87 (s, 1H), 6.68 (d, 1H, *J* = 7.6 Hz), 5.50 (d, 1H, *J* = 8.5 Hz), 4.97 (dd, 1H, *J* = 12.7 and 5.2 Hz), 4.32 (dt, 3H, *J* = 17.5 and 10.4 Hz), 4.20 (dt, 1H, *J* = 13.6 and 6.1 Hz), 3.72 (s, 3H), 3.38 (dd, 1H, *J* = 15.0 and 5.2 Hz), 3.31 (dd, 1H *J* = 14.9 and 5.3 Hz), 1.82 (m, 1H), 0.97 (d, 1H, *J* = 6.7 Hz), 0.90 (d, 6H, *J* = 6.8 Hz) ^13^C NMR (75, MHz, CDCl_3_) 172.1 (CO), 170.3 (CO), 168.3 (CO), 156.6 (C), 144.1 (2C), 141.3 (2C), 138.9 (C), 136.1 (C), 132.8 (CH), 127.7 (2CH), 127.5 (C), 127.1 (2CH), 127.1 (2CH), 127.0 (CH), 125.3 (CH), 125.2 (CH), 123.2 (CH), 122.8 (CH), 122.3 (CH), 121.4 (C), 120.0 (2CH), 119.8 (CH), 118.4 (C), 111.4 (CH), 109.6 (C), 67.3 (CH_2_), 61.4 (CH), 53.4 (CH), 52.6 (CH_3_), 47.3 (CH), 31.3 (CH), 27.3 (CH_2_), 19.4 (CH_3_), 17.6 (CH_3_).

#### 3.4.3. Synthesis of (1*S*,4*S*)-4-(1*H*-Indol-3-ylmethyl)-1-isopropyl-2*H*-pyrazino[2,1-*b*]quinazolin-3,6-(1*H*, 4*H*)-dione (**4a**)

To a solution of **vi-a** (290 mg, 0.440 mmol) in dried CH_2_Cl_2_ (20 mL) was added Ph_3_P (576 mg, 2.2 mmol, 5 equiv), I_2_ (448 mg, 2.16 mmol. 4.9 equiv), and *N*,*N*-diisopropylethylamine (774 µL, 4.44 mmol, 10 equiv). The reaction mixture was stirred at room temperature for 5 h, quenched with aqueous Na_2_CO_3_, and extracted with CH_2_Cl_2_ (3 × 100 mL), dried with Na_2_SO_4_, filtered and concentrated. Hexane was added to remove an excess of Ph_3_P, the precipitate was filtered and was treated with CH_2_Cl_2_ (10 mL) and piperidine (2.5 mL, 20%) at room temperature for 20 min, followed by solvent evaporation to provide the solid which was triturated with hexane (1 × 200 mL), CH_2_Cl_2_/PhMe (1 × 200 mL), and hexane (1 × 200 mL). The vacuum-dried crude residue was dissolved in CH_3_CN (10 mL) in the presence of DMAP (64 mg, 0.53 mmol) and refluxed for 19 h. The reaction mixture was purified by preparative TLC (EtOAc: MeOH: CH_2_Cl_2_, 50:2.5:47.5) to afford **4a** (45.5 g, 28%); m.p.: 112.8–114.1 °C, ${\left[\alpha \right]}_{D}^{30}$ = +300.55 (*c* 0.061, CHCl_3_), IR *v_max_* (KBr) 3417, 3068, 1683, 1594, 1471, 1387, 1333 cm^−1^; ^1^H NMR see Table 2; ^13^C NMR see Table 3; *m*/*z* (rel. intensity, %): 386.3 (M^+^, 8), 341.0 (5), 315 (10), 282.0 (8), 257.1 (55), 241.9 (7), 217.1 (17), 186.1 (10), 171.0 (18), 130.1 (100), 103.0 (14), 77.0 (16); (+)-HRESIMS *m*/*z* 387.1810 (M + H)^+^ (calculated for C_23_H_22_N_4_O_2_, 387.1776).

#### 3.4.4. Synthesis of *N*-[9*H*-Fluoren-9-ylmethoxy)carbonyl]-l-methylpentanyl-2*H*-aminobenzoyl-l-tryptophan methyl ester (**vi**-**b**)

To a solution of compound **iv-a** (129 mg, 0.382 mmol) in dried CH_2_Cl_2_ (10 mL) was added *N*-Fmoc-l-leucine-Cl [27] (**v-b**, 171 mg, 0.458 mmol). The mixture was stirred for 30 min, followed by addition of aqueous Na_2_CO_3_ (1 M, 7.6 mL, 7.6 mmol). After being stirred for a total 3 h, the mixture was extracted with CH_2_Cl_2_ (4 × 100 mL), dried with Na_2_SO_4_, filtered, and concentrated. The residue was purified by flash chromatography (eluent 5% MeOH in CH_2_Cl_2_) to give **vi-b** as a white solid (231.7 mg, 92%), m.p.: 193.7–194.9 °C, ${\left[\alpha \right]}_{D}^{30}$ = +18.42 (*c* 0.398, CHCl_3_), IR *v_max_* (KBr) 3405, 1744, 1695, 1586 cm^−1^; ^1^H NMR (300, MHz, CDCl_3_): 11.4 (s, 1H), 8.59 (d, 1H, *J* 8.3 Hz), 8.19 (s, 1H), 7.76 (d, 2H, *J* 7.4 Hz), 7.65 (d, 1H, *J* 7.3 Hz), 7.59 (d, 1H, *J* 7.4 Hz), 7.52-7.28 (m, 8H), 7.17 (t, 1H, *J* 7.3 Hz), 7.06 (d, 1H, *J* 7.4 Hz), 7.00 (d, 1H, 7.7 Hz) 6.96 (s, 1H), 6.73 (d, 1H, *J* 7.6 Hz), 5.55 (d, 1H, *J* 8.5 Hz), 5.05 (dd, 1H, *J* 12.6 and 5.2 Hz), 4.39 (d, 2H, *J* 6.6 Hz), 4.28 (m, 2H), 3.72 (s, 3H), 3.38 (m, 2H), 2.33 (dt, 1H *J* 13.0 and 6.4 Hz), 1.06 (d, 3H, *J* 6.8 Hz), 0.98 (d, 3H, *J* 6.8 Hz); ^13^C NMR (75, MHz, CDCl_3_): 172.1 (CO), 170.2 (CO), 168.3 (CO), 156.5 (CO), 143.8 (2C), 141.3 (2C), 139.0 (C), 136.1 (C), 132.9 (CH), 127.7 (2CH), 127.5 (C), 127.1 (2CH), 127.1 (2CH), 126.9 (CH), 125.3 (CH), 125.2 (CH), 123.2 (CH), 122.8 (CH), 122.4 (CH), 121.4 (C), 120.2 (2CH), 119.8 (CH), 118.5 (C), 111.4 (CH), 109.7 (CH), 67.2 (CH_2_), 61.3 (CH), 53.3 (CH), 52.6 (CH_3_), 38.6 (CH), 47.3 (CH_2_), 31.4 (CH), 27.3 (CH_2_), 19.4 (CH_3_), 17.5 (CH_3_).

#### 3.4.5. Synthesis of (1*S*,4*S*)-4-(1*H*-Indol-3-ylmethyl)-1-isobutyl-2*H*-pyrazino[2,1-*b*]quinazolin-3,6-(1*H*,4*H*)-dione (**5a**)

To a solution of **vi-b** (232 mg, 0.344 mmol) in dried CH_2_Cl_2_ (20 mL) was added Ph_3_P (451 mg, 1.72 mmol, 5 equiv), I_2_ (428 mg, 1.68 mmol. 4.9 equiv), and *N*,*N*-diisopropylethylamine (605 µL, 3.47 mmol, 10 equiv). The reaction mixture was stirred at room temperature for 5 h, quenched with aqueous Na_2_CO_3_, and extracted with CH_2_Cl_2_ (3 × 100 mL), dried with Na_2_SO_4_, filtered and concentrated. Hexane was added to remove an excess of Ph_3_P, the precipitate was filtered and was treated with CH_2_Cl_2_ (10 mL) and piperidine (2.5 mL, 20%) at room temperature for 20 min, followed by solvent evaporation to provide the solid which was triturated with hexane (1 × 200 mL), CH_2_Cl_2_/PhMe (1 × 200 mL), and hexane (1 × 200 mL). The vacuum-dried crude residue was dissolved in CH_3_CN (10 mL) in the presence of DMAP (80 mg, 0.66 mmol) and refluxed for 19 h. The reaction mixture was purified by preparative TLC (EtOAc:MeOH:CH_2_Cl_2_, 50:2.5:47.5) to afford **5a** (39 mg, 28%); m.p.: 105.9–106.3 °C, ${\left[\alpha \right]}_{D}^{30}$ = +81.76 (*c* 0.106, CHCl_3_), IR *v_max_* (KBr) 3435, 3060, 1686, 1602, 1387, 1292 cm^−1^; ^1^H NMR see Table 2; ^13^C NMR see Table 3; (+)-HRESIMS *m*/*z* 401.1933 (M + H)^+^ (calculated for C_24_H_24_N_4_O_2_, 401.1933).

### 3.5. General Condition for the Synthesis Compound ***4d*** and ***5d***

#### 3.5.1. Synthesis of *N*-(2-Aminobenzoyl)-d-tryptophan methyl ester (**iv-b**)

To a mixture of anthranilic acid (287 mg, 2.39 mmol) and TBTU (920 mg, 2.86mmol, 1.2 equiv) in CH_3_CN (20 mL) was added Et_3_N (833 µL, 4.78 mmol, 2 equiv) and D-tryptophan methyl ester (521 mg, 2.39 mmol) at room temperature with stirring. After being stirred for 5 h, the reaction mixture was concentrated under reduced pressure. The residue was dissolved in CH_2_Cl_2_ and washed with 1M HCl, extracted with CH_2_Cl_2_ (3 × 100 mL), dried with Na_2_SO_4_, filtered and concentrated. The residue was purified by flash chromatography (eluent: 1% MeOH in CH_2_Cl_2_) to yield **iv-b** as a white solid (569.7 mg, 81%), m.p. 131.9–134.3 °C, IR *v_max_* (KBr) 3423, 1746, 1644 cm^−1^, ^1^H NMR (300, MHz, CDCl_3_): 8.14 (s, 1H), 7.56 (d, 1H, *J* = 7.9 Hz), 7.35 (d, 1H, *J* = 8.1 Hz), 7.19–7.15 (m, 3H), 7.10 (ddd, 1H, *J* = 8.0, 7.1, and 1.1 Hz), 7.01 (d, 1H, *J* = 2.4 Hz), 6.65 (dd, 1H, *J* = 8,7 and 1.1), 6.60 (s, 1H), 6.59–6.52 (ddd, 2H, *J* = 7.5 and 0.8 Hz), 5.08 (dt, 1H, *J* = 7.5 and 5.3 Hz), 3.72 (s, 3H), 3.43 (m, 2H); ^13^C NMR (75, MHz, CDCl_3_): 172.6 (CO), 168.8 (CO), 148.8 (C), 136.1 (C), 132.6 (CH), 127.6 (CH), 127.5 (C), 122.8 (CH), 122.3 (CH), 119.8 (CH), 118.7 (CH), 117.3 (CH), 116.7 (CH), 115.3 (C), 111.3 (CH), 110.1 (C), 53.1 (CH), 52.5 (CH_3_), 27.7 (CH_2_).

#### 3.5.2. Synthesis of *N*-[9*H*-Fluoren-9-ylmethoxy)carbonyl]-d-valinyl-2-aminobenzoyl-d-tryptophan methyl ester (**vi**-**c**)

To a solution of **iv-b** (140 mg, 0.416 mmol) in dried CH_2_Cl_2_ (10 mL) was added *N*-Fmoc-d-valine-Cl [27] (**v-c**,182 mg, 0.5 mmol). The mixture was stirred for 30 min, followed by addition of aqueous Na_2_CO_3_ (1 M, 8 mL, 8 mmol). After continuous stirring for 3 h, the mixture was extracted with CH_2_Cl_2_ (4 × 100 mL), dried with Na_2_SO_4_, filtered, and concentrated. The residue was purified by flash chromatography (eluent: 5% MeOH in CH_2_Cl_2_ to give **vi-c** as a white solid (220.4 mg, 84%), m.p.: 197.8–200.2 °C, ${\left[\alpha \right]}_{D}^{30}$ = −22.72 (*c* 0.088, CHCl_3_), IR *v_max_* (KBr) 3423, 1724, 1670, 1589 cm^−1^; ^1^H NMR (300, MHz, CDCl_3_): 11.42 (s, 1H), 8.59 (d, 1H, *J* = 8.3 Hz), 8.18 (s, 1H), 7.76 (d, 2H, *J* = 7.4 Hz), 7.66 (d, 1H, *J* = 7.3 Hz), 7.60 (d, 1H, *J* = 7.3 Hz); 7.53–7.28 (m, 9H), 7.17 (t, 1H, *J* = 7.3 Hz), 7.08 (d, 1H, *J* = 7.4 Hz), 7.02 (m, 1H), 6.96 (s, 1H), 6.72 (d, 1H, *J* = 7.6 Hz), 5.55 (d, 1H, *J* = 8.5 Hz), 5.06 (dd, 1H, *J* = 12.5 and 5.2 Hz), 4.40 (d, 1H, *J* = 6.6 Hz), 4.32–4.24 (m, 1H), 3.73 (s, 3H), 3.45–3.31 (m, 2H), 1.65 (s, 3H) 2.40–2.31 (m, 1H), 1.06 (d, 3H, *J* = 6.8 Hz), 0.98 (d, 3H, *J* = 6.9 Hz); ^13^C NMR (75, MHz, CDCl_3_) 172.0 (CO), 170.2 (CO), 168.3 (CO), 156.5 (C), 144.1 (2C), 141.3 (2C), 139.0 (C), 136.1 (C), 132.9 (CH), 127.7 (2CH), 127.5 (C), 127.1 (2CH), 127.1 (2CH), 126.9 (CH), 125.3 (CH), 125.2 (CH), 123.2 (CH), 122.8 (CH), 122.4 (CH), 121.4 (C), 120.2 (2CH), 119.8 (CH), 118.5 (C), 111.4 (CH), 109.7 (C), 67.2 (CH_2_), 61.3 (CH), 53.3 (CH), 52.6 (CH_3_), 47.3 (CH), 31.3 (CH), 27.3 (CH_2_), 19.4 (CH_3_), 17.6 (CH_3_).

#### 3.5.3. Synthesis of (1*R*,4*R*)-4-(1*H*-Indol-3-ylmethyl)-1-isopropyl-2*H*-pyrazino[2,1-*b*]quinazolin-3,6-(1*H*,4*H*)-dione (**4d**)

To a solution of **vi-c** (183 mg, 0.278 mmol) in dried CH_2_Cl_2_ (20 mL) was added Ph_3_P (365 mg, 1.4 mmol, 5 equiv), I_2_ (345 mg, 1.36 mmol, 4.9 equiv), and *N*,*N*-diisopropylethylamine (489 µL, 2.81 mmol, 10 equiv). The reaction mixture was stirred at room temperature for 5 h, quenched with aqueous Na_2_CO_3_, and extracted with CH_2_Cl_2_ (3 × 100 mL), dried with Na_2_SO_4_, filtered, and concentrated. Hexane was added to remove an excess of Ph_3_P, the precipitate was filtered and treated with CH_2_Cl_2_ (10 mL) and piperidine (2.5 mL, 20%) at room temperature for 20 min, followed by solvent evaporation to provide the solid which was triturated with hexane (1 × 200mL), CH_2_Cl_2_/PhMe (1 × 200 mL), and hexane (1 × 200 mL). The vacuum-dried crude residue was dissolved in CH_3_CN (10 mL in the presence of DMAP (64 mg, 0.53 mmol) and refluxed for 19 h. The reaction mixture was purified by preparative TLC (EtOAc:MeOH:CH_2_Cl_2_, 50:2.5:47.5) to afford **4d** (22.4 mg, 21%), m.p.: 111.9–113.0 °C, ${\left[\alpha \right]}_{D}^{30}$ = −210.53 (*c* 0.114, MeOH), IR *v_max_* (KBr) 3321, 3068, 1683, 1593, 1471, 1387, 1291 cm^−1^; ^1^H NMR see Table 2; ^13^C NMR see Table 3; *m*/*z* (rel. intensity, %): 385.9 (M^+^, 12), 257.1 (29), 214.1 (3), 202.0 (7), 171.1 (10), 143.1 (4), 130.1 (100), 103.0 (18), 77 (16); (+)-HRESIMS *m*/*z* 389.1776 (M + H)^+^ (calculated for C_23_H_22_N_4_O_2_, 387.1809).

#### 3.5.4. Synthesis of *N*-[9*H*-Fluoren-9-ylmethoxy)carbonyl]-d-methylpentyl-2-aminobenzoyl-d-tryptophan methyl ester (**vi**-**d**)

To a solution of **iv-b** (130 mg, 0.386 mmol) in dried CH_2_Cl_2_ (10 mL) was added *N*-Fmoc-d-leucine-Cl [27] (**v-d**,172.5 mg, 0.464 mmol). The mixture was stirred for 30 min, followed by addition of aqueous Na_2_CO_3_ (1 M, 7.7 mL, 7.7 mmol). After continuous stirring for 3 h, the mixture was extracted with CH_2_Cl_2_ (4×), dried with Na_2_SO_4_, filtered, and concentrated. The residue was purified by flash chromatography (eluent: 5% MeOH in CH_2_Cl_2_) to give **vi-d** as a white solid (251 mg, 98%), m.p.: 194.9–196.3 °C, ${\left[\alpha \right]}_{D}^{30}$ = −31.75 (*c* 0.105, CHCl_3_), IR *v_max_* (KBr) 1740, 1645, 1584 cm^−1^; ^1^H NMR (300, MHz, CDCl_3_): 11.5 (s, 1H), 8.58 (d, 1H, *J* = 8.3 Hz), 8.23 (s, 1H), 7.76 (d, 2H, *J* = 7.4 Hz), 7.66 (d, 1H, *J* = 7.4 Hz), 7.57 (d, 1H, *J* = 7.4 Hz), 7.51–7.26 (m, 8H), 7.16 (t, 1H, *J* = 7.4 Hz), 7.07 (d, 1H, *J* = 7.4 Hz), 7.00 (t, 1H, *J* = 7.5 Hz) 6.93 (s, 1H), 6.74 (d, 1H, *J* = 7,6 Hz), 5.43 (d, 1H, *J* = 7.9 Hz), 5.04 (dd, 1H, *J* = 12.7 and 5.2 Hz), 4.41 (dt, 3H, *J* = 17.5 and 10.4 Hz), 4.25 (t, 1H, *J* = 7.0 Hz), 3.72 (s, 3H), 3.38 (dd, 1H, *J* = 15.0 and 5.2 Hz), 3.31 (dd, 1H *J* = 14.9 and 5.3 Hz), 1.82 (m, 1H), 1.00 (d, 6H, *J* = 6.2 Hz); ^13^C NMR (75 MHz, CDCl_3_): 172.1 (CO), 170.2 (CO), 168.3 (CO), 156.4 (CO), 143.8 (2C), 141.3 (2C), 139.0 (C), 136.1 (C), 132.9 (CH), 127.7 (2CH), 127.5 (C), 127.1 (2CH), 127.1 (2CH), 126.9 (CH), 125.3 (CH), 125.2 (CH), 123.2 (CH), 122.8 (CH), 122.4 (CH), 121.4 (C), 120.2 (2CH), 119.8 (CH), 118.5 (C), 111.4 (CH), 109.7 (CH), 67.2 (CH_2_), 61.3 (CH), 53.3 (CH), 52.6 (CH_3_), 47.3 (CH_2_), 38.6 (CH), 31.4 (CH), 27.3 (CH_2_), 19.4 (CH_3_), 17.5 (CH_3_).

#### 3.5.5. Synthesis of (1*R*,4*R*)-4-(1*H*-Indol-3-ylmethyl)-1-isobutyl-2*H*-pyrazino[2,1-*b*]quinazolin-3,6-(1*H*,4*H*)-dione (**5d**)

To a solution of **vi-d** (251 mg, 0.373 mmol) in dried CH_2_Cl_2_ (20 mL) was added Ph_3_P (489 mg, 1.9 mmol, 5 equiv), I_2_ (464 mg, 1.83 mmol, 4.9 equiv), and *N*,*N*-diisopropylethylamine (656 µL, 3.77 mmol, 10 equiv). The reaction mixture was stirred at room temperature for 5 h, quenched with aqueous Na_2_CO_3_, and extracted with CH_2_Cl_2_ (3 × 100 mL), dried with Na_2_SO_4_, filtered, and concentrated. Hexane was added to remove an excess of Ph_3_P, the precipitate was filtered and was treated with CH_2_Cl_2_ (10 mL) and piperidine (2.5 mL, 20%) at room temperature for 20 min, followed by solvent evaporation to provide the solid which was triturated with hexane (1 × 200 mL), CH_2_Cl_2_/PhMe (1 × 200 mL), and hexane (1 × 200 mL). The vacuum-dried crude residue was dissolved in CH_3_CN (10 mL in the presence of DMAP (84 mg, 0.82 mmol) and refluxed for 19 h. The reaction mixture was purified by preparative TLC (EtOAc:MeOH:CH_2_Cl_2_, 50:2.5:47.5) to afford **5d** (61.6 mg, 40%), m.p.: 103.2–105.6 °C, ${\left[\alpha \right]}_{D}^{30}$ = −186.04 (*c* 0.086, CHCl_3_), IR *v_max_* (KBr) 3333, 3061, 1687, 1603, 1296 cm^−1^; ^1^H NMR see Table 2; ^13^C NMR see Table 3; (+)-HRESIMS *m*/*z* 401.1966 (M + H)^+^ (calculated for C_24_H_24_N_4_O_2_, 401.1933).

### 3.6. Screening Test for Antitumor and Anti-P-Glycoprotein Activity

Compounds **4a**–**d** and **5a**–**d** were reconstituted in sterile DMSO to the final concentration of 60 mM, and several aliquots were made and stored at −20 °C to avoid repeated freeze-thaw cycles. For experiments, the compounds were freshly diluted in medium to the desired concentration. Screening for tumor cell growth inhibition was carried out in two human tumor cell lines (NCI-H460 and HCT-15), with the sulforhodamine B (SRB) assay, as previously described [30]. Briefly, tumor cells were plated in 96-well plates, incubated at 37 °C for 24 h, and then treated for 48 h with 5 serial dilutions (1:2) of each compound (ranging from 150 μM to 9.375 μM). The effect of the vehicle solvent (DMSO) was also analyzed as a control. Cells were fixed with 10% ice-cold trichloroacetic acid, washed with water and stained with SRB. Finally, the plates were washed with 1% acetic acid and the bound SRB was solubilized with 10 mM Tris Base. Absorbance was measured in a microplate reader (Synergy Mx, Biotek Instruments Inc., Winooski, VT, USA) at 510 nm. For each compound, the corresponding GI_50_ (concentration which inhibited 50% of net cell growth) was determined, as previously described [33]. For the screening of compounds for drug-efflux inhibitory activity, the flow cytometry determination of rhodamine-123 cellular accumulation was carried out as previously described [34]. Briefly, K562 and K562Dox cells were incubated for 1 h at 37 °C with 20 μM of the compounds, and 1 μM of rhodamine-123 (Rh123, from Sigma, USA). Verapamil was used as a positive control. Cells were then washed, resuspended in ice cold PBS, and analyzed in a BD Accuri™ C6 Flow Cytometer (BD Biosciences, San Jose, CA, USA). Data were analyzed using the FlowJo software (version 7.6.1, Tree Star, Inc.). The ratio of Rh123 accumulation in the cells was then calculated as MFI_K562Dox+Compound_ -MFI_K562Dox_)/MFI_K562Dox_ [30].

## 4. Conclusions

Inspired by the marine-derived fiscalin B (**4c**), quinazolinone alkaloid derivatives were synthesized using two different methodologies: a highly efficient and straightforward three-component one-pot microwave-assisted approach and also a multistep Mazurkiewicz–Ganesan approach. While the former proved to be efficient and practical for broad screening libraries of the compounds, the latter, although with a more intricate methodology, proved to be a good approach for the synthesis of the syn enantiomers. Moreover, we have found that partial epimerization under the reaction conditions could occur. In vitro growth inhibitory activity of two tumor cell lines revealed that among this series of synthesized compounds, six new analogues were found to exhibit tumor cell growth inhibitory activity. Consequently, this marine-inspired synthesis can bring new insights into discovery of new lead compounds in the oncology area.

## Figures and Tables

**Table 1:**
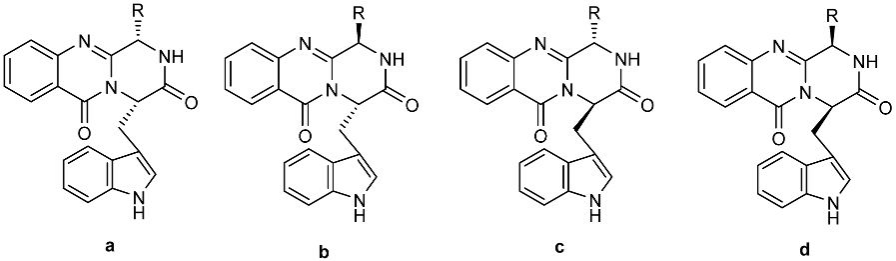
Enantiomers and diastereomers of the pyrazinoquinazolinone alkaloids **4** and **5**.

Entry	R ^a^	Tryptophan	% *Anti* Compound	% *Syn* Compound	[α]_D_^b^	Enantiomeric Ratio ^c^	Purity (%) ^d^
**1**	l-valine	l-tryptophan	10 (**4b**)	-	+63.8	40:60	87
**2**	d-valine	l-tryptophan	14 (**4b**)	-	+65.2	39:61	89
**3**	l-valine	d-tryptophan	8 (**4c**)	-	−248.1	62:37	90
**4**	d-valine	d-tryptophan	10 (**4c**)	-	−100.0	63:37	91
**5**	l-leucine	l-tryptophan	7 (**5b**)	-	+44.9	67:33	94
**6**	d-leucine	l-tryptophan	10 (**5b**)	-	+89.7	50:50	94
**7**	l-leucine	d-tryptophan	8 (**5c**)	-	−61.7	31:69	90
**8**	d-leucine	d-tryptophan	10 (**5c**)	-	−142.9	46:54	86
9 *	l-valine	l-tryptophan	-	28 (**4a**)	+300.5	100:0	94
10 *	d-valine	d-tryptophan	-	21(**4d**)	−210.5	7:93	89
11 *	l-leucine	l-tryptophan	-	28 (**5a**)	+81.8	90:10	89
12 *	d-leucine	d-tryptophan	-	40 (**5d**)	−186.0	17:83	85

^a^ R residual of amino acid at C-1 position; ^b^ Optical rotation, concentration (g/100 mL); ^c^ Calculated from the peak area from chiral HPLC experiments (by using equation X × 100/Xn in which X is the peak area of each peak and Xn is the total peak area); ^d^ Calculated from the peak area from reversed-phase HPLC experiments;* Entry of Mazurkiewicz–Ganesan approach.

**Table 2:** ^1^H NMR data (300 MHz, CDCl_3_) for **4** and **5**.

Position	δ_H_ (*J* in Hz)			
	4a	4b	4c	4d
H-1	3.95, dd (8.4,3.5)	2.69, d (2.4)	2.69, d (2.4)	3.95, dd (8.5, 3.6)
H-2	6.50, d (3.0)	5.85, br	5.80, br	6.39, d (3.2)
H-4	5.52, dd (6.3, 3.8)	5.68, dd (5.0, 2.8)	5.67, dd (5.0, 2.8)	5.52, dd (6.3, 3.8)
H-8	8. 38, dd (8.0, 1.1)	8. 37, dd (8.0, 1.1)	8. 37, dd (8.0, 1.1)	8. 38, dd (8.0, 1.2)
H-9	7.54, ddd (8.2, 7.2, 1.2)	7.54, t (3.0)	7.54, t (5.2)	7.54, ddd (8.1, 7.3, 1.1)
H-10	7.79, ddd (8.7, 7.7, 1.9)	7.77, ddd (7.9, 7.7, 1.9)	7.77, ddd (8.9, 6.2, 1.9)	7.79, ddd (8.5, 7.1, 1.2)
H-11	7.62, d (7.7)	7.57, d (3.4)	7.57, d (3.5)	7.62, d (7.7)
H-1′	1.06–0.94, m	2.66–2.61, m	2.66–2.61, m	1.06–0.94, m
H-2′	0.48, d (6.6)	0.63, d (2.3)	0.63, d (4.0)	0.48, d (6.6)
H-3′	0.75, d (6.8)	0.66, d (3.7)	0.65, d (4.3)	0.75, d (6.8)
H-4′a	3.72, dd (14.9, 3.7)	3.69, dd (15.0, 5.3)	3.69, dd (17.8, 4.0)	3.72, dd (14.8, 3.7)
H-4′b	3.80, dd (14.9, 6.3)	3.77, dd (15.0, 2.9)	3.77, dd (15.0, 2.8)	3.80, dd (14.8, 6.4)
H-6′	6.89, d (2.4)	6.60, d (2.3)	6.60, d (2.4)	6.90, d (2.3)
H-7′	8.06, br	8.03, br	8.03, br	8.03, br
H-9′	7.28, d (8.4)	7.28, d (8.2)	7.28, d (8.2)	7.28, d (7.8)
H-10′	7.10, ddd (8.6, 7.6, 1.0)	7.13, t (7.1)	7.13, t (7.1)	7.10, ddd (8.5, 7.5 0.9)
H-11′	6.93, ddd (8.0, 7.1, 1.0)	6.93, t (7.5)	6.93, t (7.5)	6.94, ddd (8.2, 7.2, 1.0)
H-12′	7.49, d (7.9)	7.44, d (8.0)	7.44, d (8.1)	7.49, d (7.9)
	**5a**	**5b**	**5c**	**5d**
H-1	4.32, dt (10.7, 3.2)	2.73, dd (9.7, 3.4)	2.72, d (9.7)	4.31, dt (11.0, 3.2)
H-2	6.45, d (2.5)	5.75, br	5.75, br	6.21, d (2.5)
H-4	5.54, dd (5.2, 3.3)	5.68, dd (5.2, 3.0)	5.68, dd (5.0, 2.9)	5.55, dd (5.2, 3.3)
H-8	8. 39, dd (8.0, 1.2)	8. 37, d (8.0)	8. 37, d (8.0)	8. 39, dd (8.0, 1.1)
H-9	7.41, d (8.0)	7.55, d (8.1)	7.55, d (8.0)	7.42, d (8.0)
H-10	7.78, ddd (8.5, 7.2, 1.5)	7.78, ddd (8.5, 7.1, 1.5)	7.78, ddd (8.5, 7.1, 1.5)	7.79, ddd (8.5, 7.1, 1.6)
H-11	7.57, d (7.8)	7.60, d (7.8)	7.60, d (7.9)	7.59, d (7.8)
H-1′	2.84–2.44, m	1.43–1.33, m	1.44–1.36, m	2.89–2.56, m
H-2′	2.08–2.00, m	2.01, ddd (12.4, 12.5, 4.2)	2.01, dd (13.0, 9.4)	2.07–2.00, m
H-3′	0.75, d (6.0)	0.77, d (6.4)	0.77, d (6.3)	0.74, d (6.0)
H-3″	0.49, d (6.1)	0.28, d (6.5)	0.28, d (6.4)	0.49, d (6.0)
H-4′a	3.75, dd (15.0, 3.3)	3.65, dd (15.0, 5.3)	3.65, dd (14.9, 5.2)	3.75, dd (15.1, 3.4)
H-4′b	3.83, dd (15.0, 5.3)	3.77, dd (15.0, 2.6)	3.78, dd (15.1, 2.8)	3.84, dd (15.0, 5.3)
H-6′	6.68, d (2.3)	6.64, d (2.3)	6.64, d (2.0)	6.68, d (2.4)
H-7′	8.08, br	8.09, br	8.07, br	8.03, br
H-9′	7.28, d (8.1)	7.29, d (8.2)	7.29, d (8.2)	7.29, d (8.3)
H-10′	7.12, ddd (8.5, 7.1, 1.1)	7.13, t (7.6)	7.13, t (8.0)	7.12, ddd (8.1, 7.0, 1.1)
H-11′	6.97, ddd (8.5, 7.0, 1.1)	6.98, t (7.5)	6.98, t (7.5)	6.93, ddd (8.0, 7.1, 1.0)
H-12′	7.53, ddd (8.1, 7.1, 1.0)	7.50, d (8.0)	7.50, d (8.0)	7.53, ddd (8.2, 7.2, 1.1)

**Table 3:** ^13^C NMR data (75 MHz, CDCl_3_) for 4 and 5.

Position	δ_C_, Type							
	4a	4b	4c	4d	5a	5b	5c	5d
C-1	61.9, CH	58.1	58.1	61.8	54.1, CH	50.8	50.8	54.1
C-3	167.8, CO	169.5	169.5	167.8	167.6, CO	169.4	169.4	167.5
C-4	57.5, CH	56.8	57.3	57.5	56.7, CH	57.3	57.3	56.7
C-6	161.4, CO	160.9	160.9	161.0	161.0, CO	160.8	160.8	161.1
C-7	120.2, C	120.2	120.2	120.2	120.4, C	120.3	120.3	120.0
C-8	126.8, CH	126.9	126.9	126.7	126.9, CH	126.9	126.9	126.8
C-9	127.1, CH	127.2	127.2	127.1	126.9, CH	127.4	127.4	126.8
C-10	134.7, CH	134.7	134.7	134.7	134.8, CH	134.6	134.6	134.7
C-11	127.1, CH	127.0	127.0	127.1	126.7, CH	127.1	127.1	126.7
C-12	146.8, C	147.1	147.1	146.1	147.2, C	147.0	147.0	147.2
C-14	149.3, C	150.3	150.3	149.8	151.3, C	151.5	151.5	151.3
C-1′	34.5, CH	29.4	29.5	34.8	45.8, CH_2_	40.2	40.2	45.8
C-2′	18.0, CH_3_	14.8	14.8	18.1	23.9, CH	24.1	24.1	23.9
C-3″	-	-	-	-	20.4, CH_3_	19.7	19.7	20.4
C-3′	19.6, CH_3_	18.8	18.8	19.8	22.9, CH_3_	23.3	23.3	22.9
C-4′	27.4, CH_2_	27.4	27.4	27.4	26.4, CH_2_	27.1	27.1	26.4
C-5′	110.2, C	109.3	109.3	110.2	107.9, C	109.7	109.7	107.9
C-6′	123.5, CH	123.6	123.6	123.6	123.6, CH	123.6	123.6	123.6
C-8′	136.0, C	136.0	136.0	135.9	137.1, C	136.1	136.1	137.1
C-9′	110.9, CH	111.1	111	111	110.2, CH	111.1	111.1	110.2
C-10′	122.3, CH	122.6	122.6	122.4	122.2, CH	122.8	122.8	122.2
C-11′	119.9, CH	120.0	120.0	119.9	119.9, CH	120.2	120.2	119.9
C-12′	118.7, CH	118.7	118.7	118.7	119.0, CH	118.8	118.8	119.1
C-13′	127.8, C	127.2	127.2	127.8	126.8, C	127.1	127.1	126.8

**Table 4:** The GI_50_ of **4a**–**d** and **5a**–**d** in the NCI-H460 and HCT-15 human tumor cell lines.

Compound	GI_50_ (µM)	
	NCI-H460	HCT-15
**4a**	81.33 ± 1.55	40.33 ± 3.12
**4b**	70.20 ± 3.15	38.15 ± 0.29
**4c**	57.62 ± 2.08	31.78 ± 1.21
**4d**	60.10 ± 2.61	33.30 ± 1.37
**5a**	32.52 ± 4.24	48.18 ± 2.51
**5b**	41.52 ± 2.52	51.94 ± 4.26
**5c**	31.19 ± 3.01	43.63 ± 0.25
**5d**	36.47 ± 3.98	47.00 ± 1.47

Values were determined with the SRB assay and are the mean ± SEM of three independent experiments. Doxorubicin was used as a positive control, with the following GI_50_ concentrations: 23.02 ± 0.54 nM in NCI-H460 cells, 331.49 ± 49 nM in HCT-15 cells.

## Supplementary Materials

The following are available online at http://www.mdpi.com/1660-3397/16/8/261/s1.
