# Supplementary material for: Antitumor Activity of Quinazolinone Alkaloids Inspired by Marine Natural Products
Source: Mar Drugs. 2018 Jul 31;16(8):261. doi: 10.3390/md16080261 (PMC6117665; doi:10.3390/md16080261)
Supplement: Supplementary file 1 [file marinedrugs-16-00261-s001.pdf]

Antitumor activity of quinazolinone alkaloids inspired by marine natural products

Solida Long [1], **Diana I. S. P. Resende** [1,2], **Anake Kijjoa** [2,3], **Artur M. S. Silva** [4], **André Pina** [5,6,7], **Tamara Fernández-Marcelo** [5,6], **M. Helena Vasconcelos** [5,6,8], **Emília Sousa** [1,2,*], and **Madalena M. M. Pinto** [1,2]

1 Laboratório de Química Orgânica e Farmacêutica, Departamento de Ciências Químicas, Faculdade de Farmácia, Universidade do Porto, Rua de Jorge Viterbo Ferreira, 228, 4050-313 Porto, Portugal. E-mail: up201502099@ff.up.pt (S.L.), madalena@ff.up.pt (M.M.M.P).

2 Interdisciplinary Centre of Marine and Environmental Research (CIIMAR), Terminal de Cruzeiros do Porto de Lexões, Av. General Norton de Matos s/n, 4450-208, Matosinhos, Portugal. E-mail: dresende@ff.up.pt.

3 *ICBAS* - Instituto de Ciências Biomédicas Abel Salazar, Universidade do Porto, Rua de Jorge Viterbo Ferreira, 228, 4050-313 Porto, Portugal. E-mail: ankijjoa@icbas.up.pt.

4 QOPNA, Department of Chemistry, University of Aveiro, 3810-193 Aveiro, Portugal. E-mail: artur.silva@ua.pt (A.S.)

5 i3S - Instituto de Investigação e Inovação em Saúde, Universidade do Porto, Portugal. E-mail: andre.fig.pina@gmail.com (A.P.), tmarcelo@ipatimup.pt (T.M.), hvasconcelos@ipatimup.pt (H.V.)

6 Cancer Drug Resistance Group, IPATIMUP - Institute of Molecular Pathology and Immunology of the University of Porto, Portugal;

7 FCUP - Faculty of Sciences of the University of Porto, Portugal;

8 Laboratório de Microbiologia, Departamento de Ciências Biológicas, Faculdade de Farmácia, Universidade do Porto, Portugal.

* Correspondence: esousa@ff.up.pt; Tel.: +351-2-2042-8689

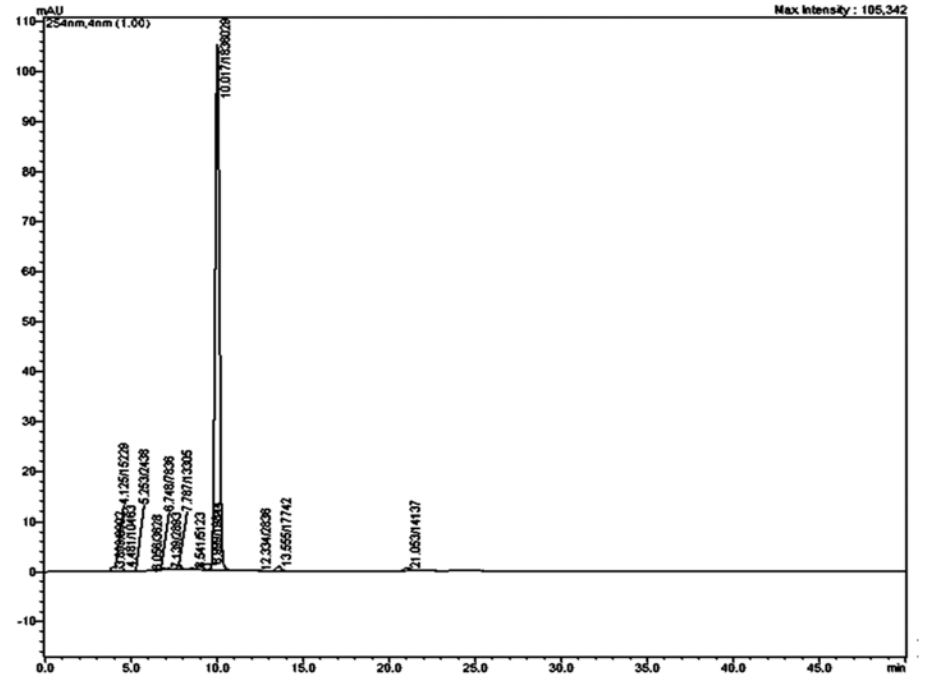

Figure S1. Peak purity analysis of compound **4a** using RP-HPLC with diode array detector (DAD), C18 column (Kimetex®, 2.6 EV0 C18 100 Å, 150 × 4.6 mm); mobile phase: methanol: water (60:40); flowrate: 1 mL/min.

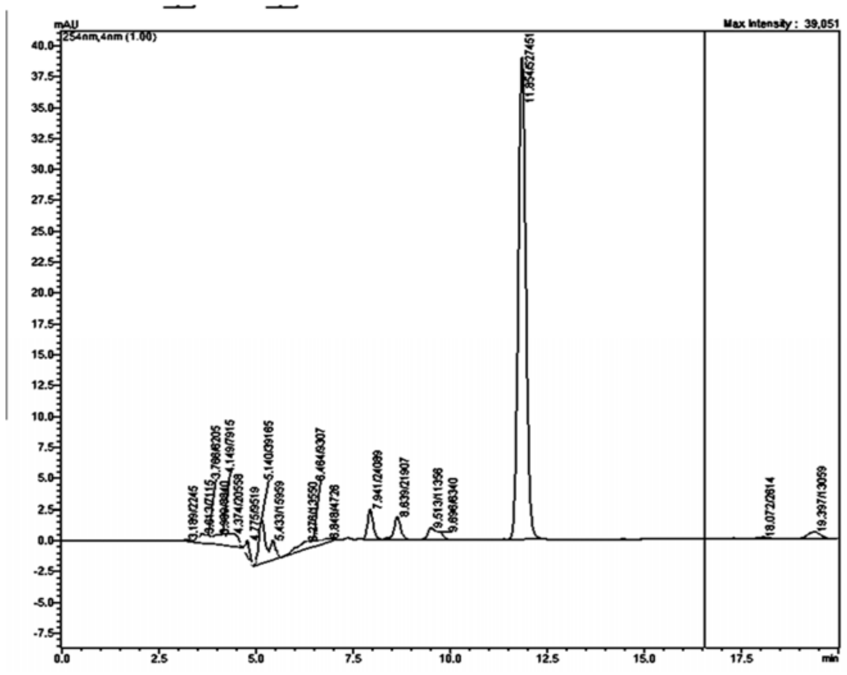

Figure S2. Peak purity analysis of compound **4b** using RP-HPLC with diode array detector (DAD), C18 column (Kimetex®, 2.6 EV0 C18 100 Å, 150 × 4.6 mm); mobile phase: methanol: water (60:40); flowrate: 0.5 mL/min.

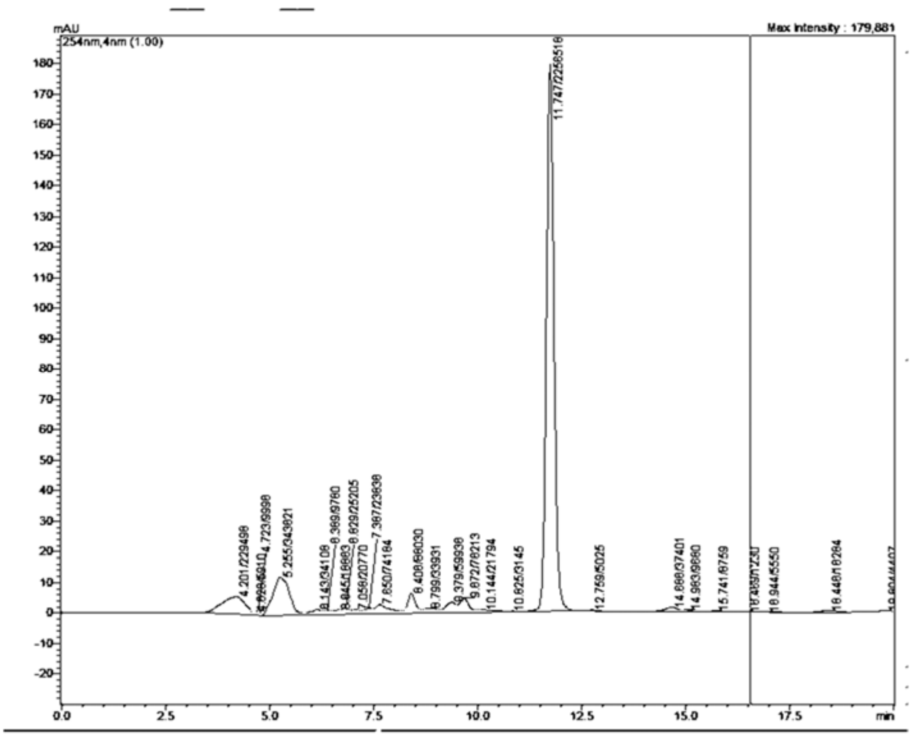

Figure S3. Peak purity analysis of compound **4c** using RP-HPLC with diode array detector (DAD), C18 column (Kimetex®, 2.6 EV0 C18 100 Å, 150 × 4.6 mm); mobile phase: methanol: water (60:40); flowrate: 0.5 mL/min.

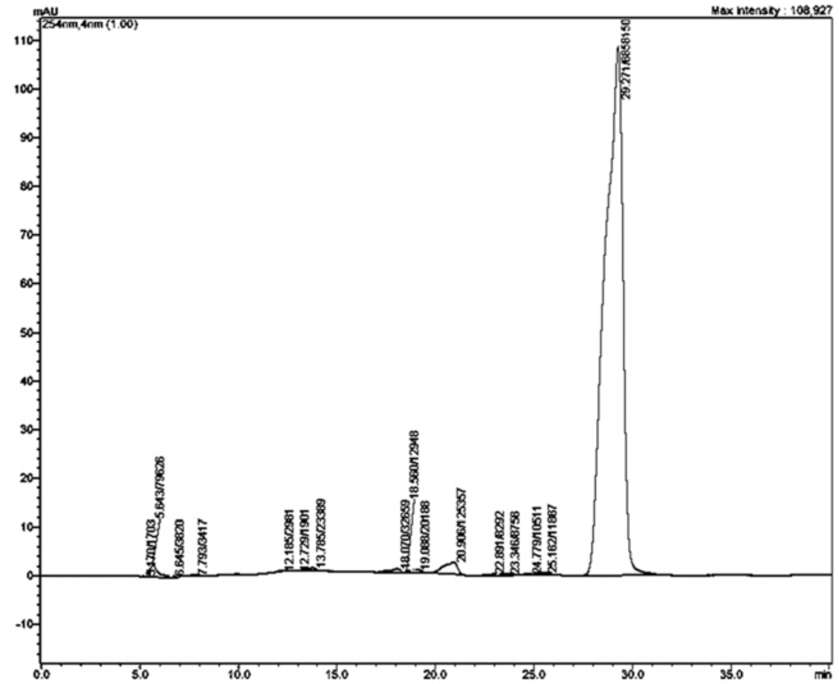

Figure S4. Peak purity analysis of compound **4d** using RP-HPLC with diode array detector (DAD), C18 column (Kimetex®, 2.6 EV0 C18 100 Å, 150 × 4.6 mm); mobile phase: methanol: water (60:40); flowrate: 0.5 mL/min.

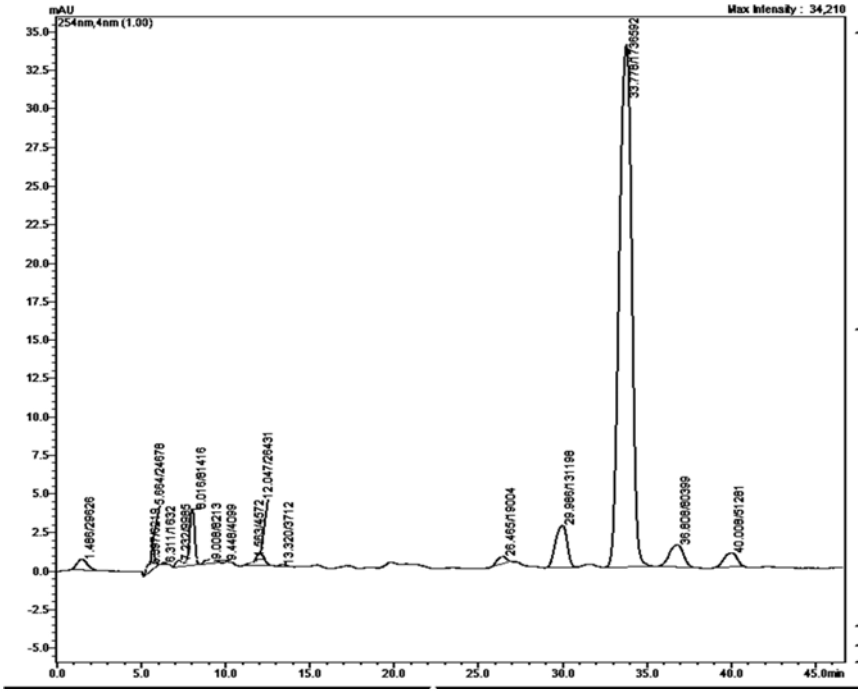

Figure S5. Peak purity analysis of compound **5a** using RP-HPLC with diode array detector (DAD), C18 column (Kimetex®, 2.6 EV0 C18 100 Å, 150 × 4.6 mm); mobile phase: methanol: water (60:40); flowrate: 0.5mL/min.

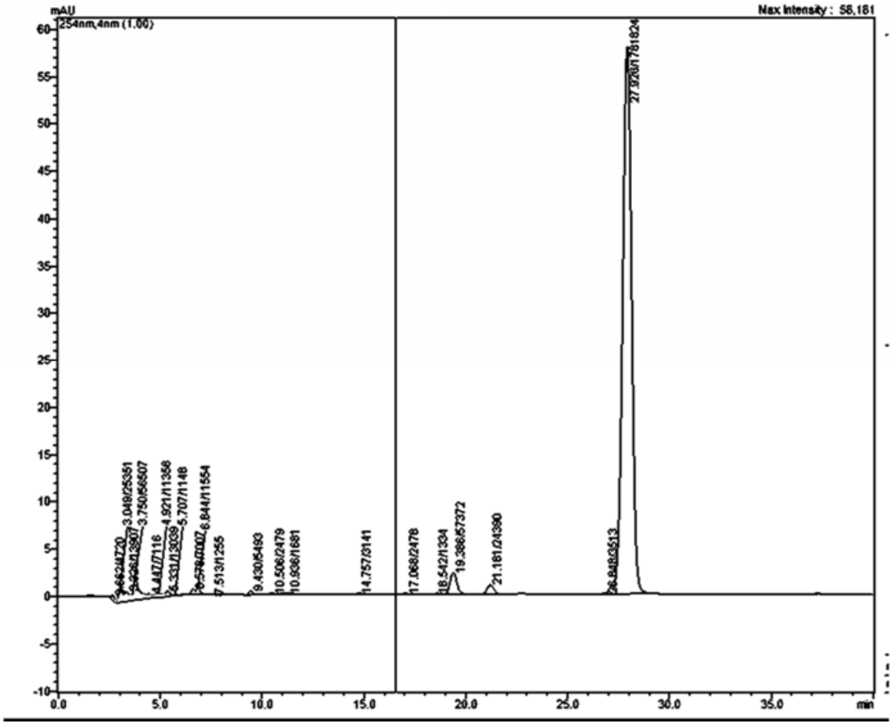

Figure S6. Peak purity analysis of compound **5b** using RP-HPLC with diode array detector (DAD), C18 column (Kimetex®, 2.6 EV0 C18 100 Å, 150 × 4.6 mm); mobile phase: methanol: water (60:40); flowrate: 0.5 mL/min.

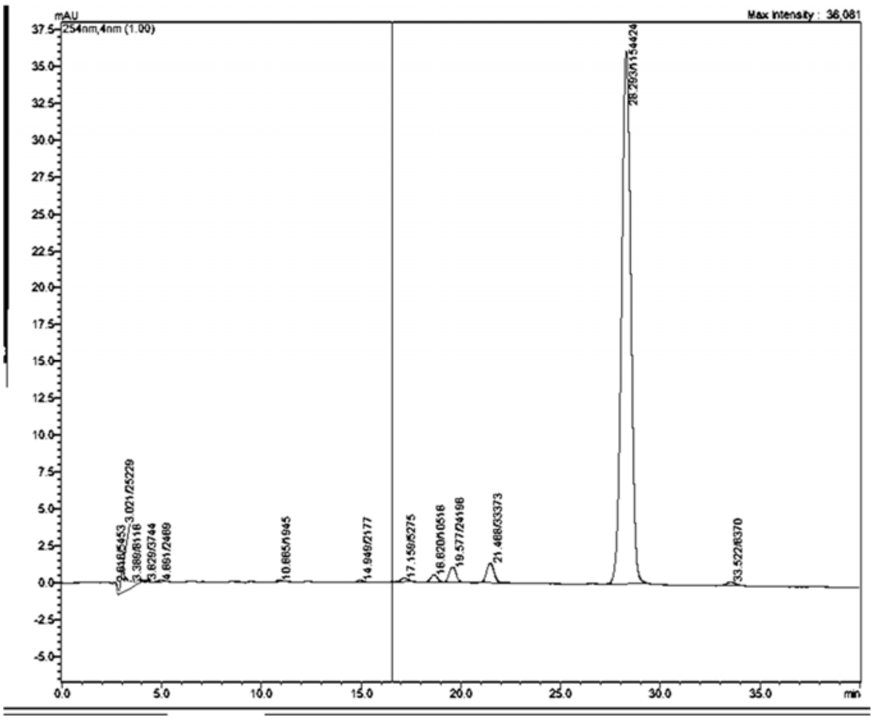

Figure S7. Peak purity analysis of compound **5c** using RP-HPLC with diode array detector (DAD), C18 column (Kimetex®, 2.6 EV0 C18 100 Å, 150 × 4.6 mm); mobile phase: methanol: water (60:40); flowrate: 0.5 mL/min.

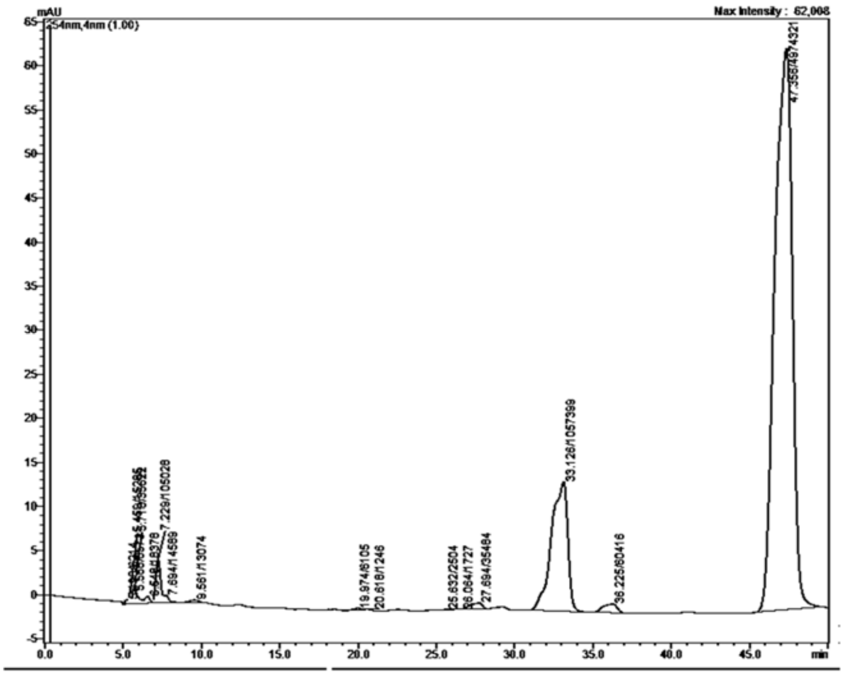

Figure S8. Peak purity analysis of compound **5d** using RP-HPLC with diode array detector (DAD), C18 column (Kimetex®, 2.6 EV0 C18 100 Å, 150 × 4.6 mm); mobile phase: methanol: water (60:40); flowrate: 0.5 mL/min.

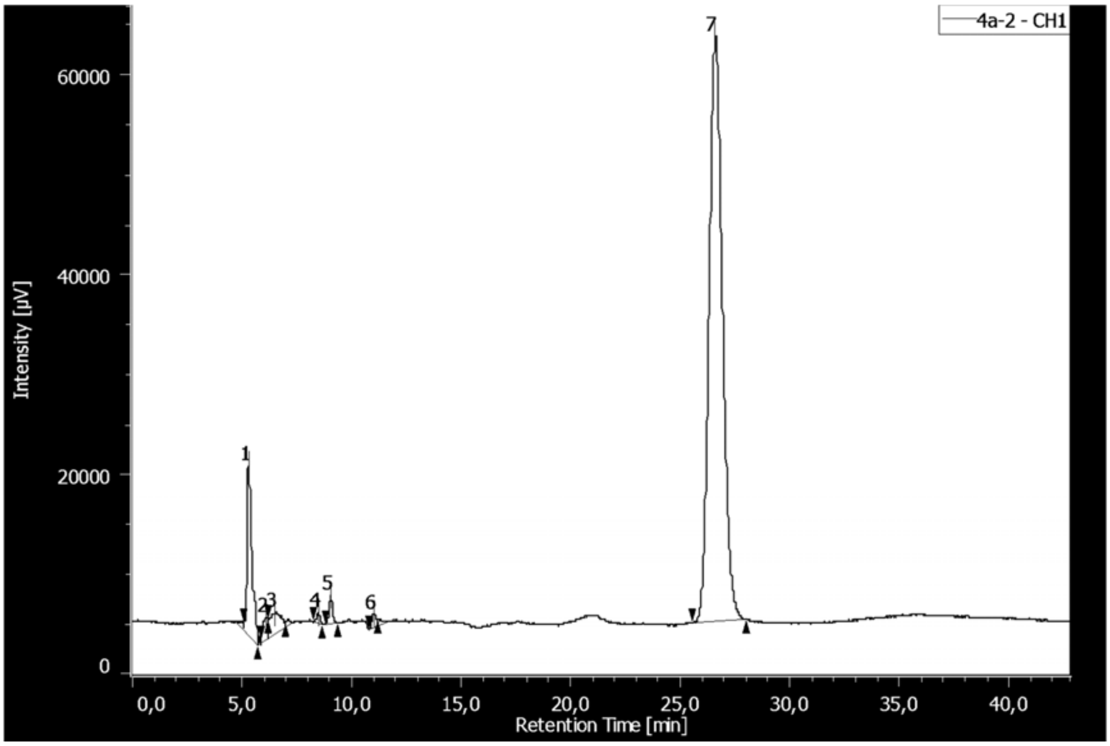

Figure S9. Chiral analysis of (1*S*,4*S*)-4-(1*H*-indol-3-ylmethyl)-1-isopropyl-2*H*-pyrazino[2,1-*b*]quinazolin-3,6-(1*H*, 4*H*)-dione (**4a**). Column: Lux® 5 µm Amylose-1, 250 × 4.6 mm; UV-detection: 254 nm; mobile phase: hexane:ethanol (80:20); flow rate: 0.5 mL/min.

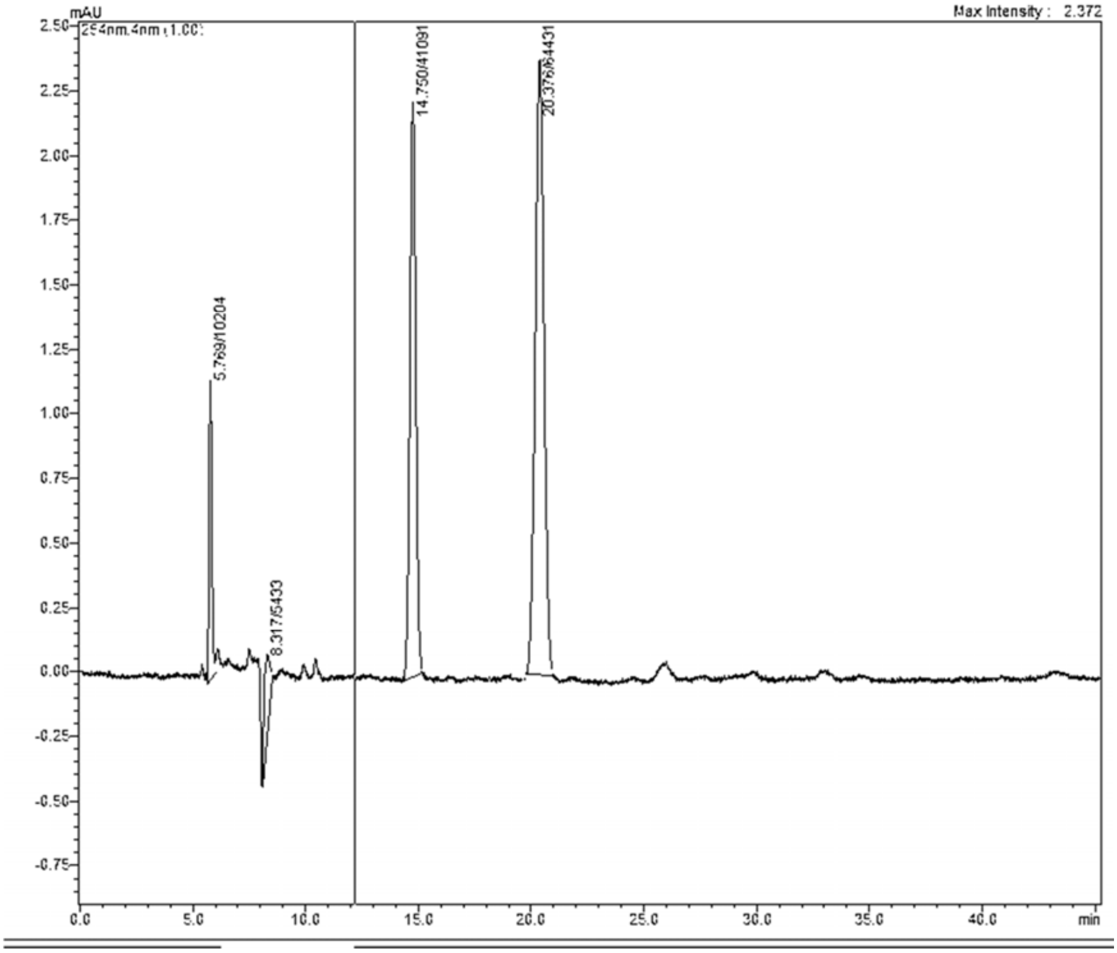

Figure S10. Chiral analysis of (1*S*,4*R*)-4-(1*H*-indol-3-ylmethyl)-1-isopropyl-2*H*-pyrazino[2,1-*b*]quinazolin-3,6-(1*H*, 4*H*)-dione (**4b**). Column: Lux® 5 µm Amylose-1, 250 × 4.6 mm; UV-detection: 254 nm; mobile phase: hexane:ethanol (80:20); flow rate: 0.5 mL/min.

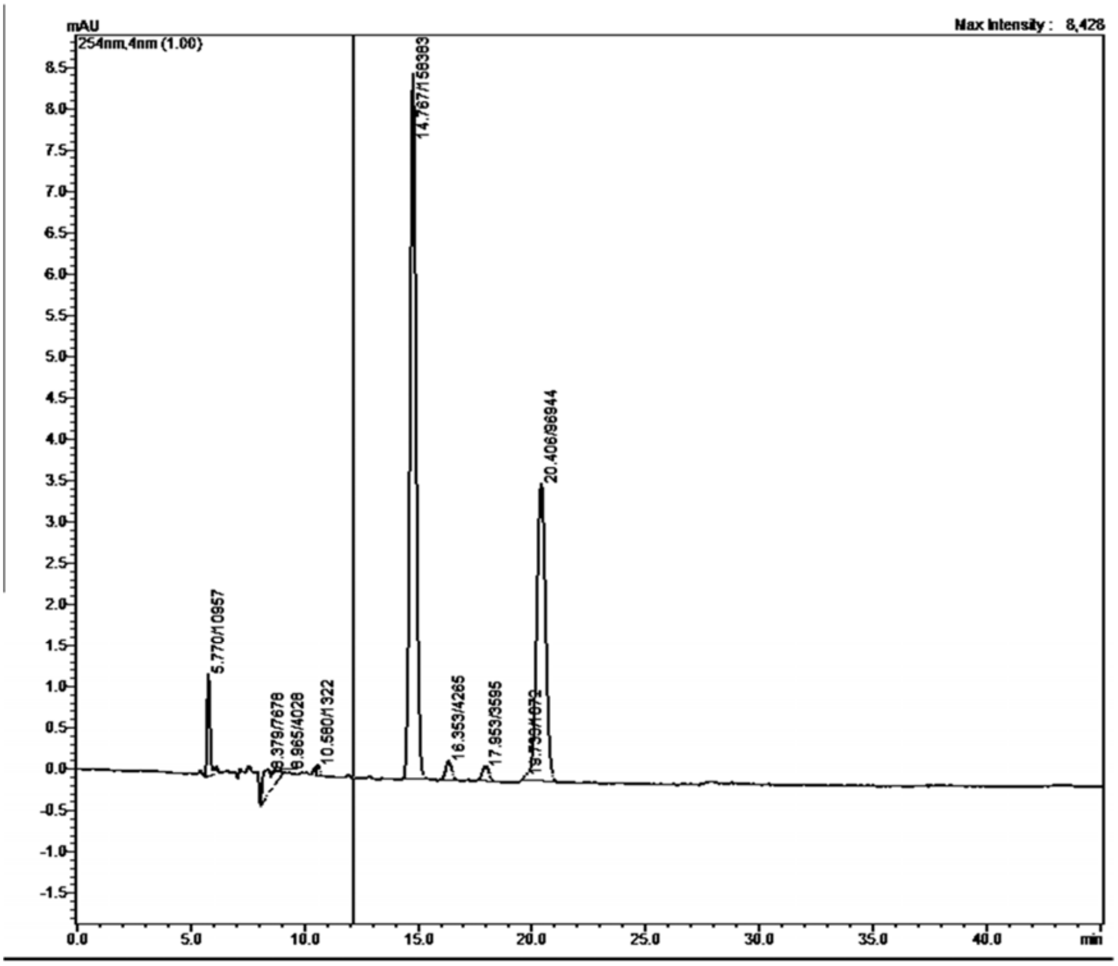

Figure S11. Chiral analysis of (1*R*,4*S*)-4-(1*H*-indol-3-ylmethyl)-1-isopropyl-2*H*-pyrazino[2,1-*b*]quinazolin-3,6-(1*H*, 4*H*)-dione (**4c**). Column: Lux® 5 µm Amylose-1, 250 × 4.6 mm; UV-detection: 254 nm; mobile phase: hexane:ethanol (80:20); flow rate: 0.5 mL/min.

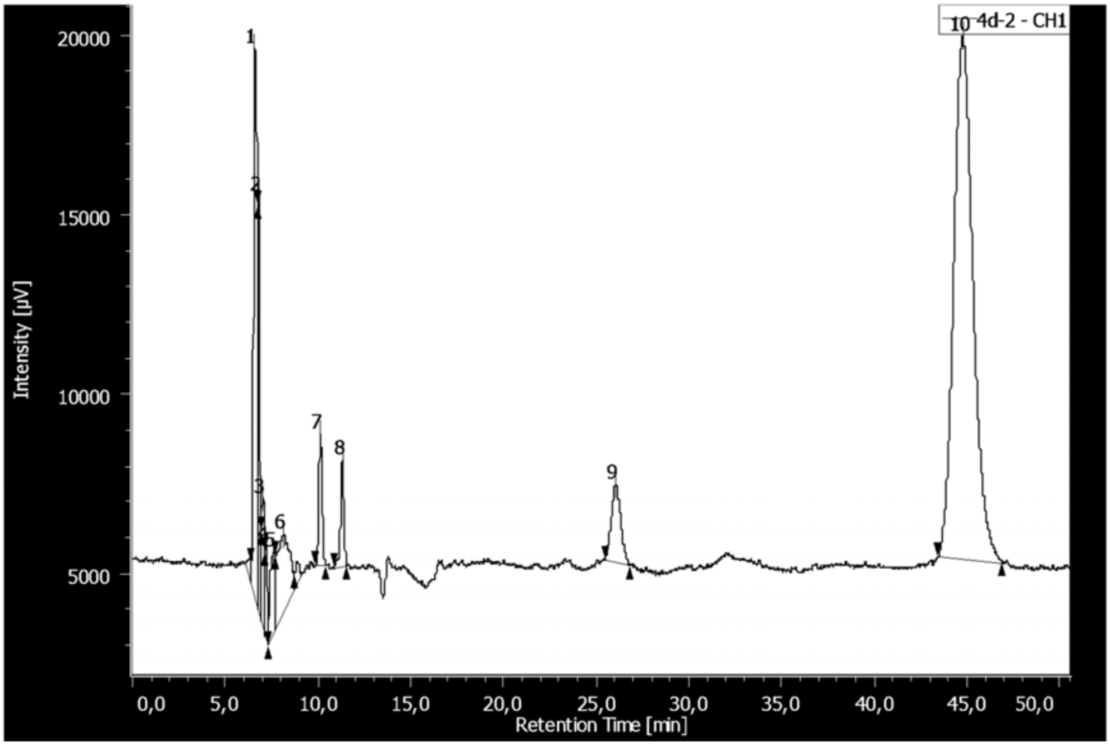

Figure S12. Chiral analysis of (1*R*,4*R*)-4-(1*H*-indol-3-ylmethyl)-1-isopropyl-2*H*-pyrazino[2,1-*b*]quinazolin-3,6-(1*H*, 4*H*)-dione (**4d**). Column: Lux® 5 µm Amylose-1, 250 × 4.6 mm; UV-detection: 254 nm; mobile phase: hexane:ethanol (80:20); flow rate: 0.5 mL/min.

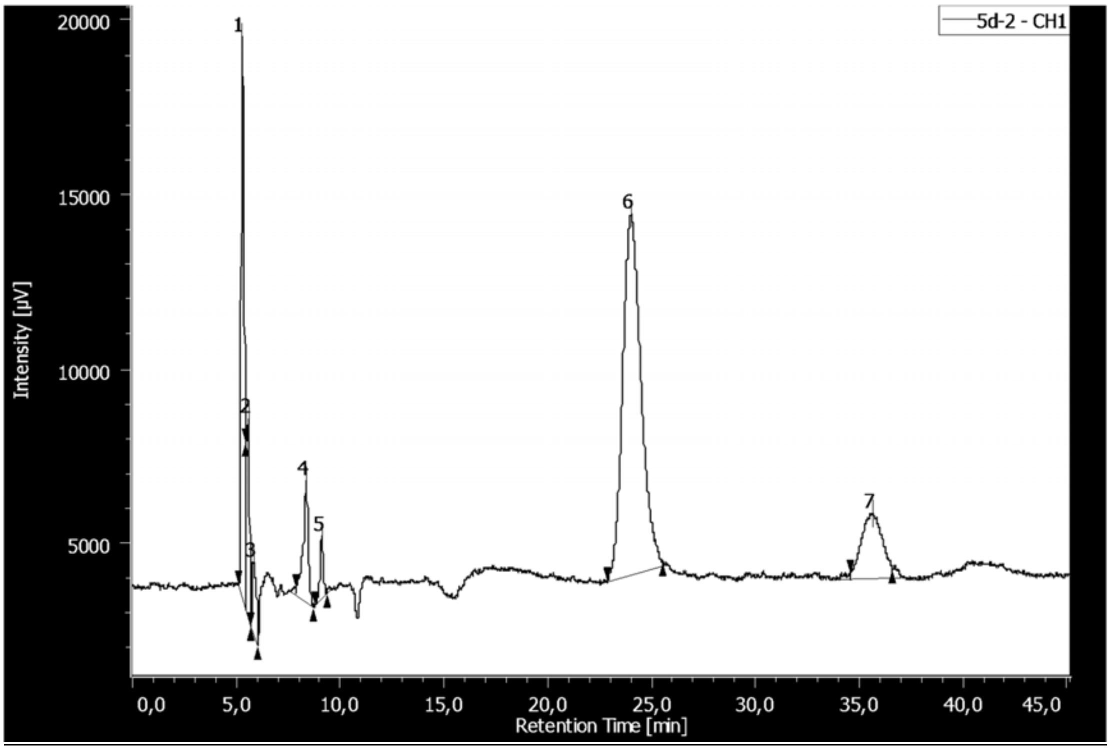

Figure S13. Chiral analysis of (1*S*,4*S*)-4-(1*H*-indol-3-ylmethyl)-1-isobutyl-2*H*-pyrazino[2,1-*b*]quinazolin-3,6-(1*H*, 4*H*)-dione (**5a**). Column: Lux® 5 µm Amylose-1, 250 × 4.6 mm; UV-detection: 254 nm; mobile phase: hexane:ethanol (80:20); flow rate: 0.5 mL/min.

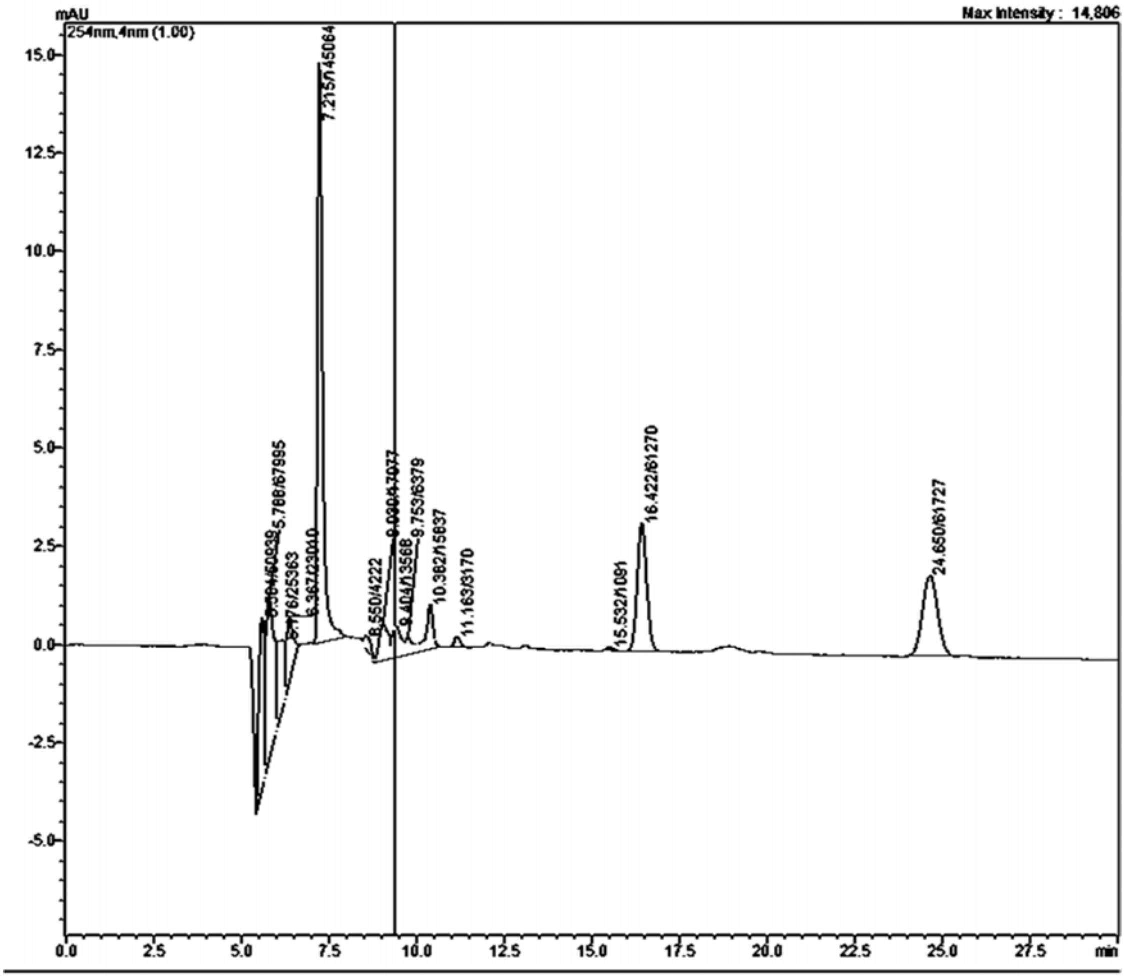

Figure S14. Chiral analysis of (1*S*,4*R*)-4-(1*H*-indol-3-ylmethyl)-1-isobutyl-2*H*-pyrazino[2,1-*b*]quinazolin-3,6-(1*H*, 4*H*)-dione (**5b**). Column: Lux® 5 µm Amylose-1, 250 × 4.6 mm; UV-detection: 254 nm; mobile phase: hexane:ethanol (80:20); flow rate: 0.5 mL/min.

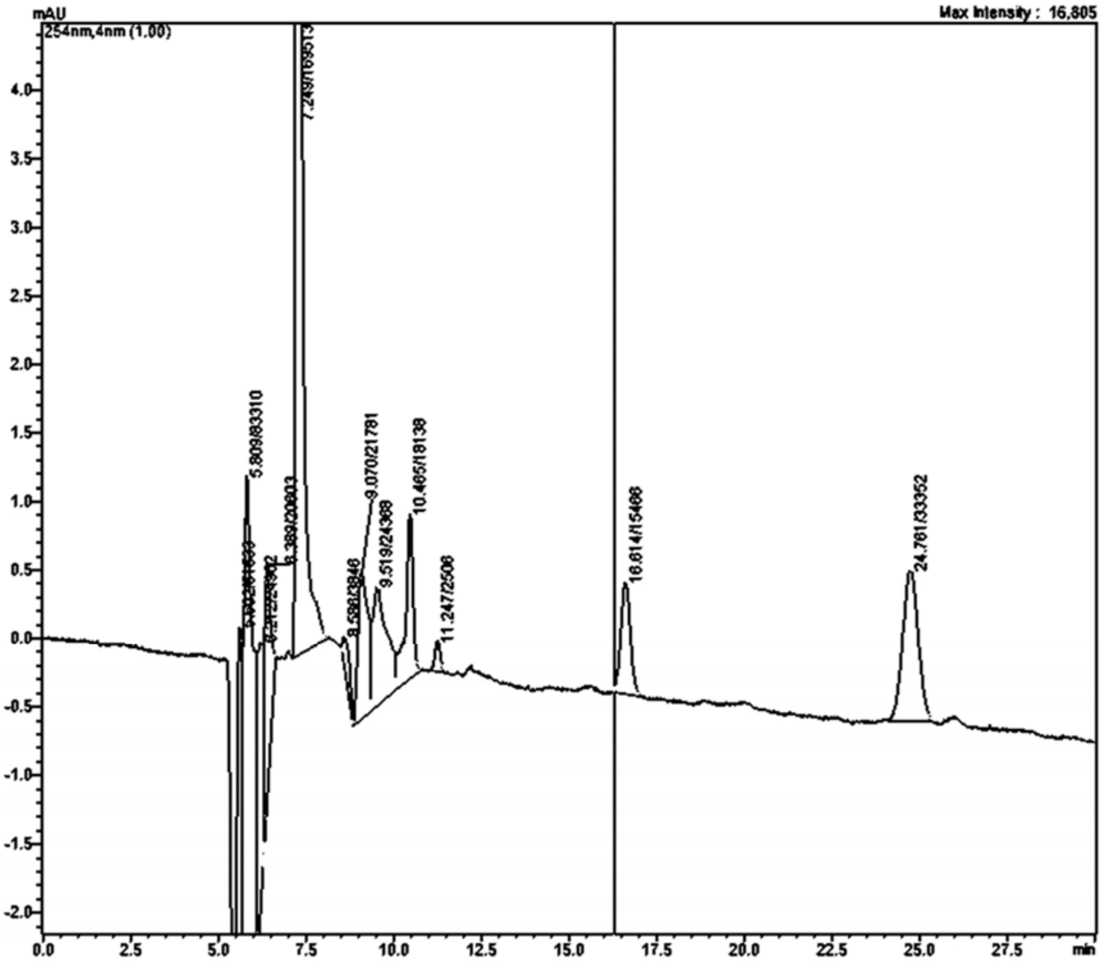

Figure S15. Chiral analysis of (1*R*,4*S*)-4-(1*H*-indol-3-ylmethyl)-1-isobutyl-2*H*-pyrazino[2,1-*b*]quinazolin-3,6-(1*H*, 4*H*)-dione (**5c**). Column: Lux® 5 µm Amylose-1, 250 × 4.6 mm; UV-detection: 254 nm; mobile phase: hexane:ethanol (80:20); flow rate: 0.5 mL/min.

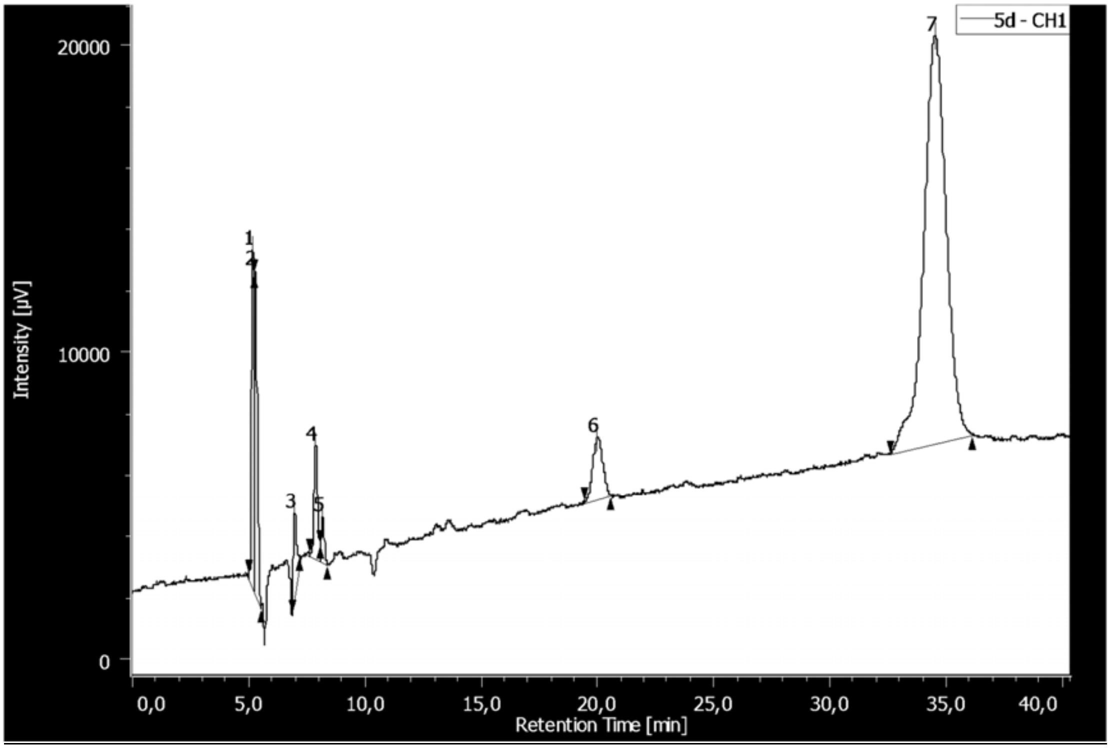

Figure S16. Chiral analysis of (1*R*,4*R*)-4-(1*H*-indol-3-ylmethyl)-1-isobutyl-2*H*-pyrazino[2,1-*b*]quinazolin-3,6-(1*H*, 4*H*)-dione (**5d**). Column: Lux® 5 µm Amylose-1, 250 × 4.6 mm; UV-detection: 254 nm; mobile phase: hexane:ethanol (80:20); flow rate: 0.5 mL/min.

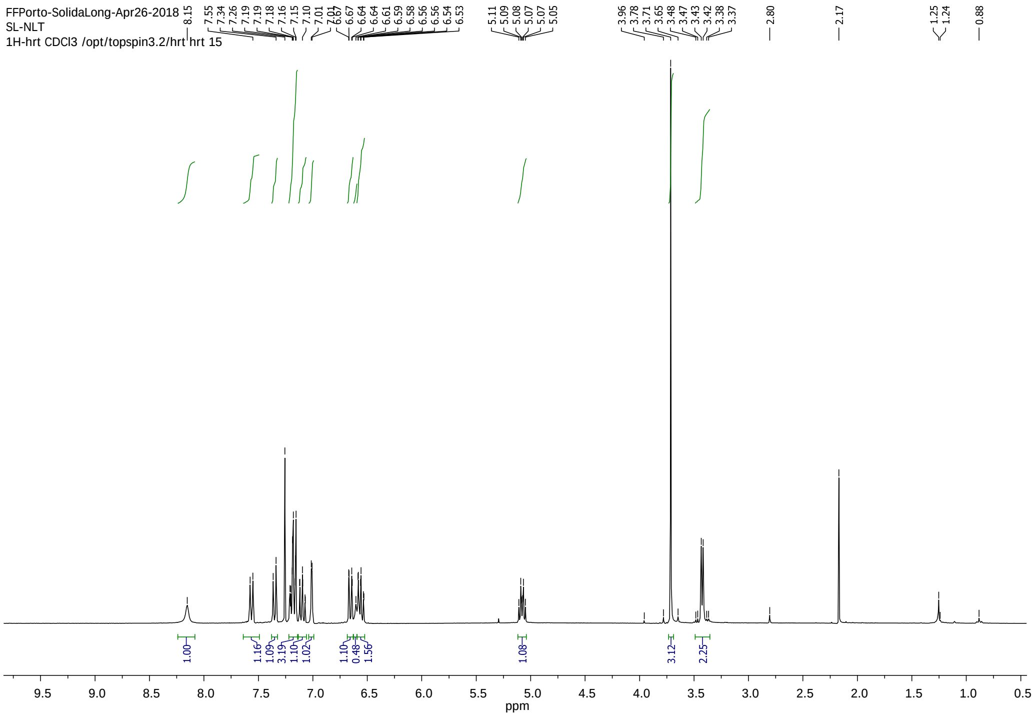

Figure S17. ^{1}H NMR spectrum of *N*-(2-aminobenzoyl)-L-tryptophan methyl ester **(iv-a)** ($CDCl_3$, 300, MHz).

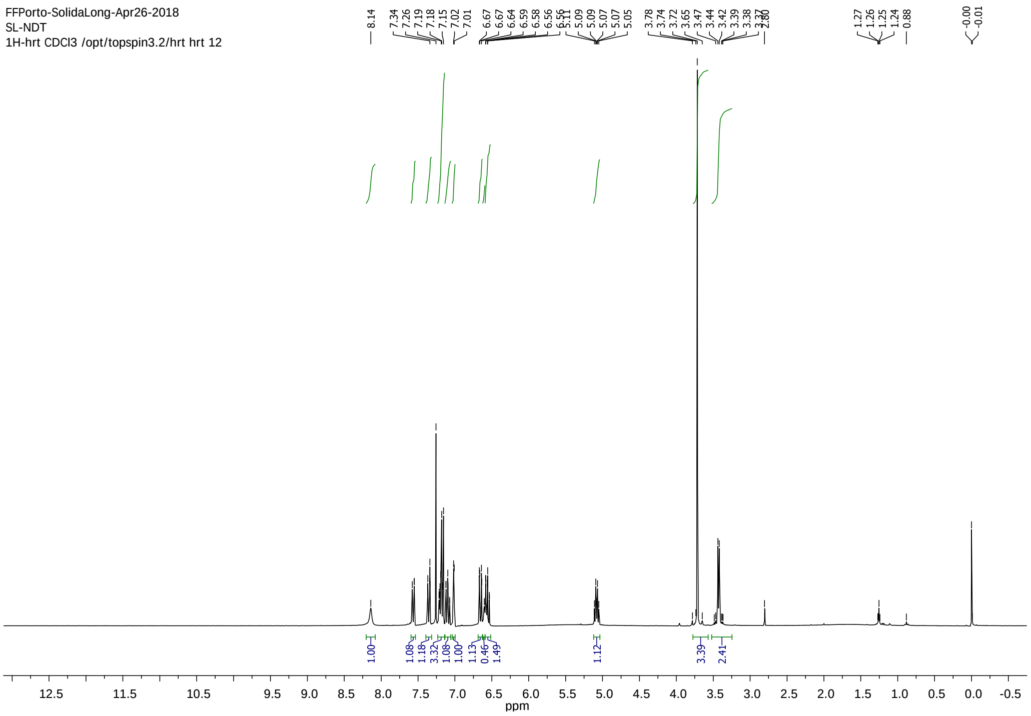

Figure S18. ^{1}H NMR spectrum of *N*-(2-aminobenzoyl)-D-tryptophan methyl ester **(iv-b)** ($CDCl_3$, 300, MHz).

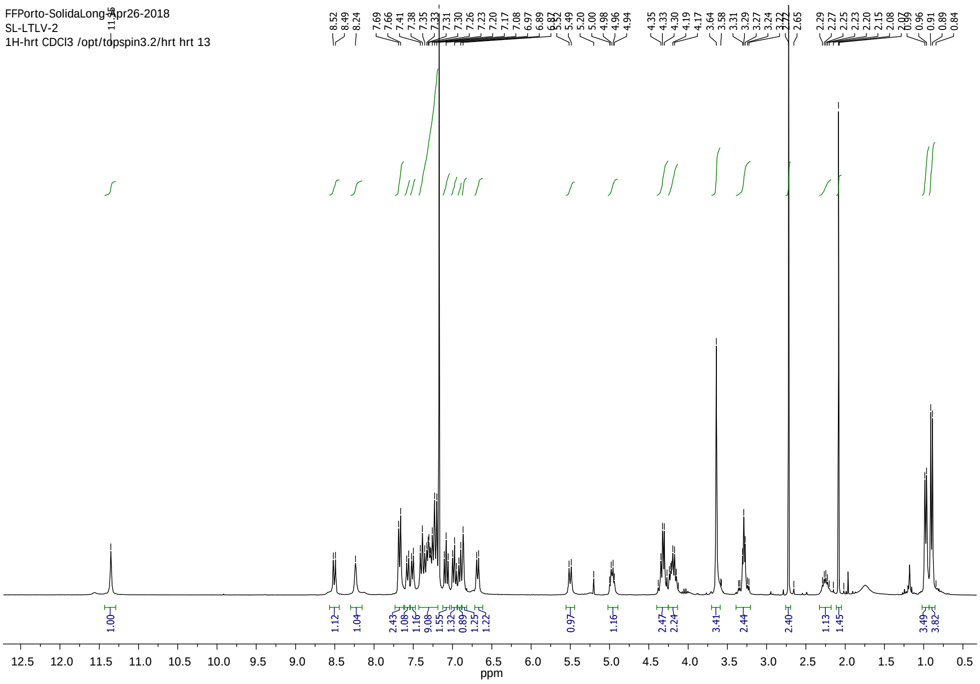

Figure S19. ^{1}H NMR spectrum of *N*-[9*H*-fluoren-9-ylmethoxy)carbonyl]-L-valinyl-2-aminobenzoyl-L-tryptophan methyl ester (**vi-a**) ($CDCl_3$, 300, MHz).

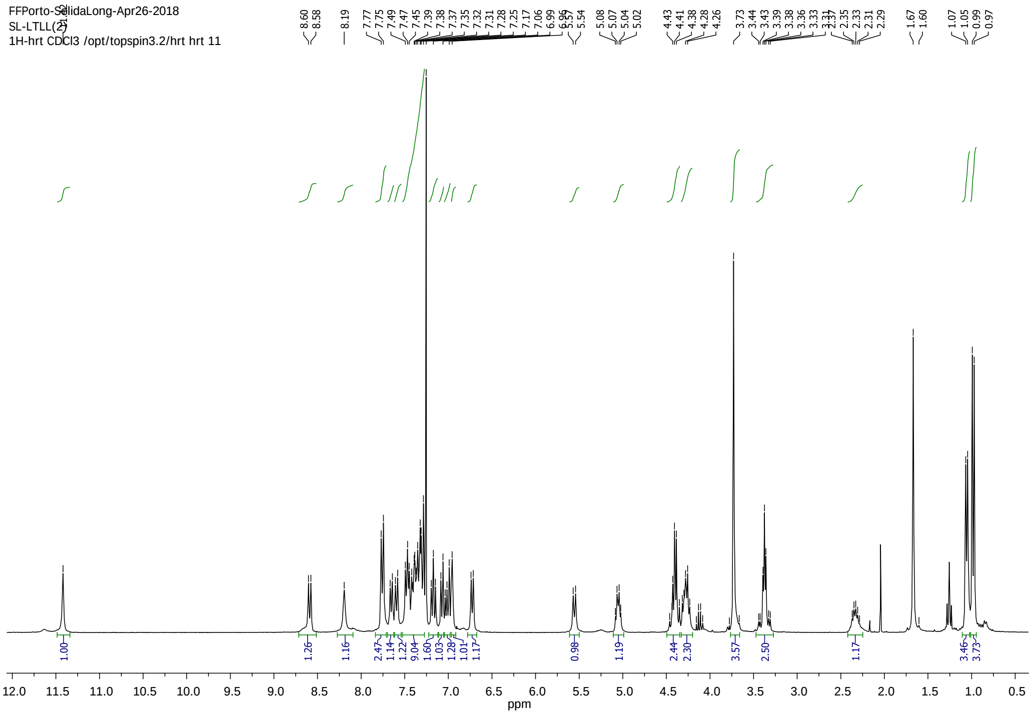

Figure S20. ^{1}H NMR spectrum of *N*-[9*H*-fluoren-9-ylmethoxy)carbonyl]-L-methylpentanyl-2*H*-aminobenzoyl-L- tryptophan methyl ester (**vi-b**) ($CDCl_3$, 300, MHz).

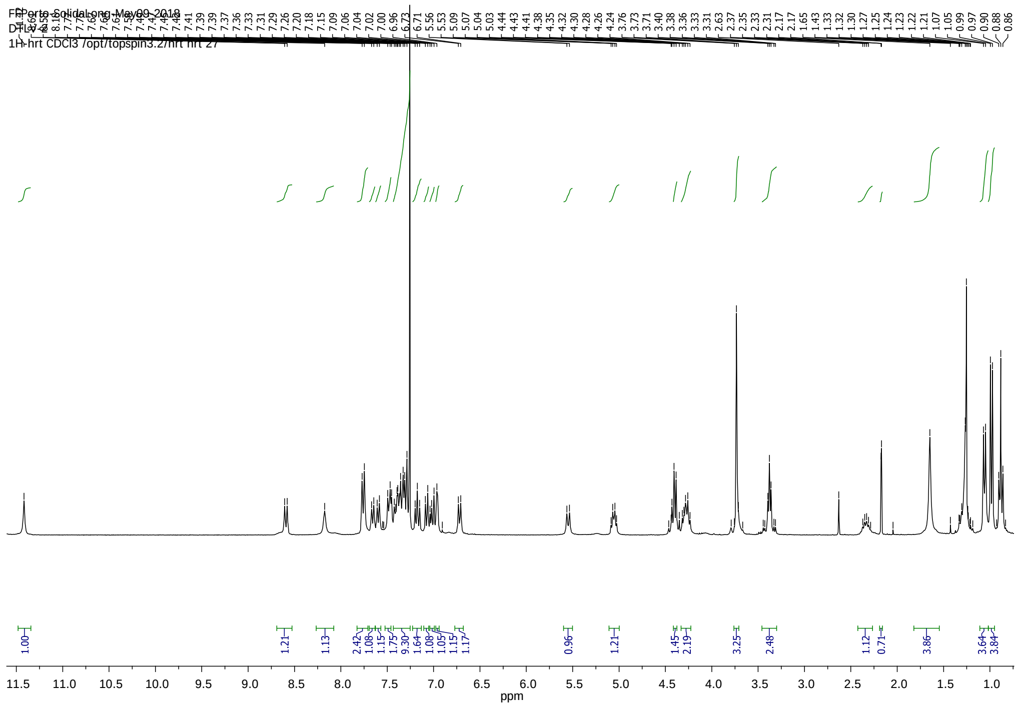

Figure S21. ^{1}H NMR spectrum of *N*-[9*H*-fluoren-9-ylmethoxy)carbonyl]-D-valinyl-2-aminobenzoyl-D-tryptophan methyl ester (**vi-c**) ($CDCl_3$, 300, MHz).

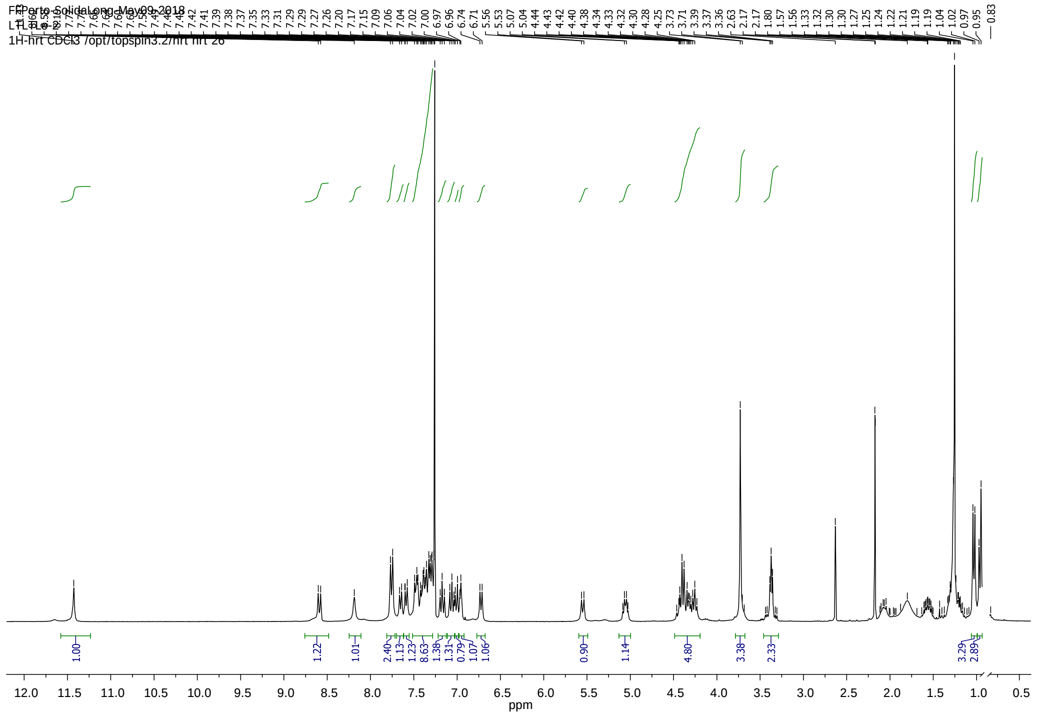

Figure S22. ^{1}H NMR spectrum of *N*-[9*H*-fluoren-9-ylmethoxy)carbonyl]-D-methylpentyl-2-aminobenzoyl-D- tryptophan methyl ester (**vi-d**) ($CDCl_3$, 300, MHz).

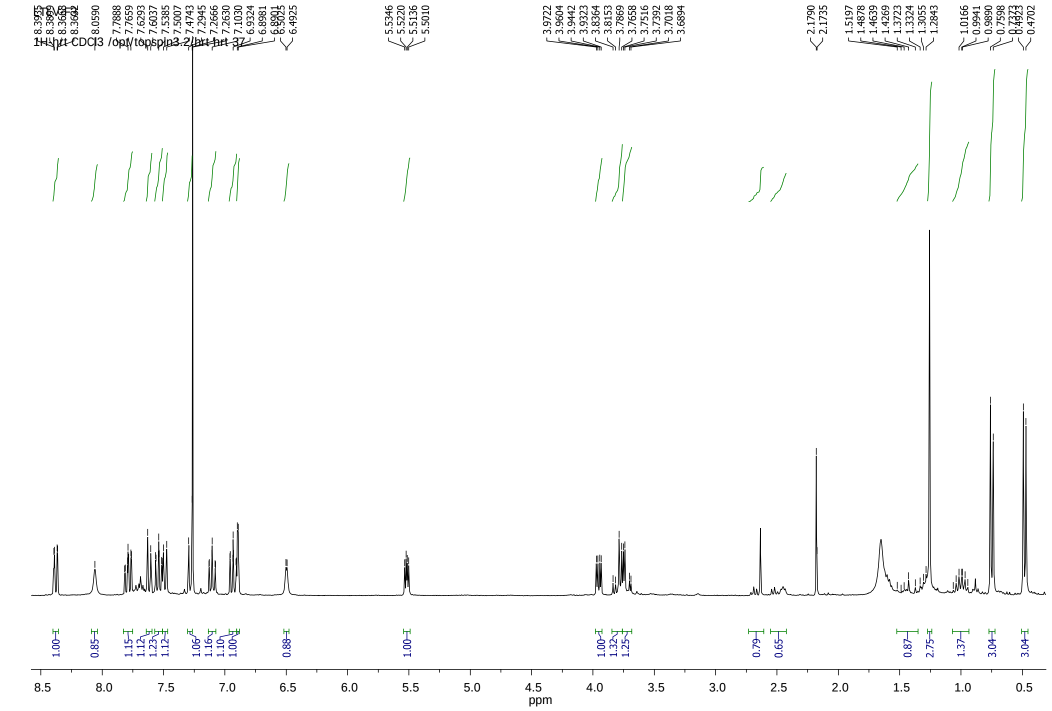

Figure S23. ^{1}H NMR spectrum of (1*S*,4*S*)-4-(1*H*-indol-3-ylmethyl)-1-isopropyl-2*H*-pyrazino[2,1-*b*]quinazolin-3,6-(1*H*, 4*H*)-dione (**4a**) ($CDCl_3$, 300, MHz).

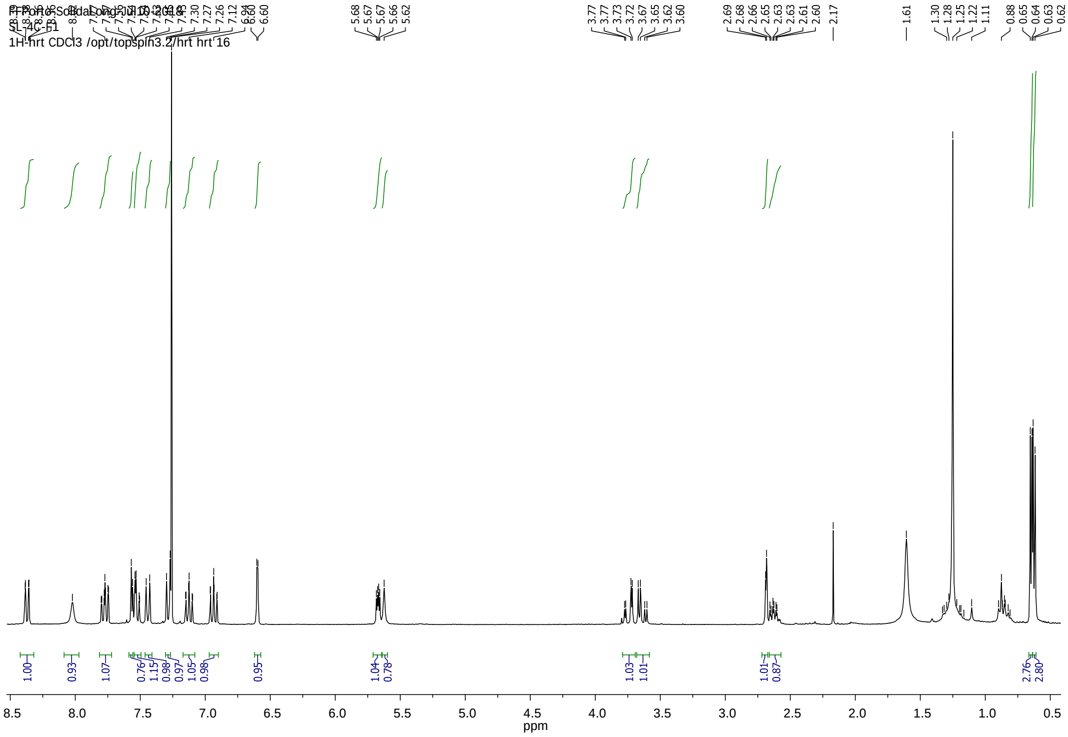

Figure S24. ^{1}H NMR spectrum of (1*R*,4*S*)-4-(1*H*-Indol-3-ylmethyl)-1-isopropyl-2*H*-pyrazino[2,1-*b*]quinazoline-3,6-(1*H*,4*H*)-dione (**4b**) ($CDCl_3$, 300, MHz).

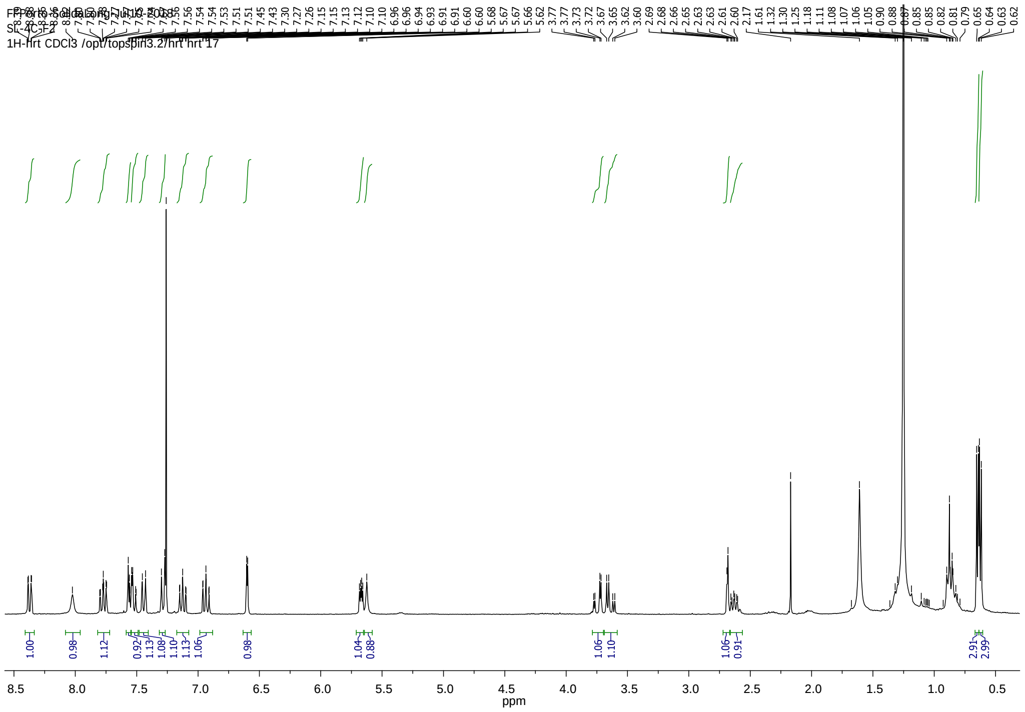

Figure S25. ^{1}H NMR spectrum of (1*S*,4*R*)-4-(1*H*-Indol-3-ylmethyl)-1-isopropyl-2*H*-pyrazino[2,1-*b*]quinazoline-3,6-(1*H*,4*H*)-dione (**4c**) ($CDCl_3$, 300, MHz).

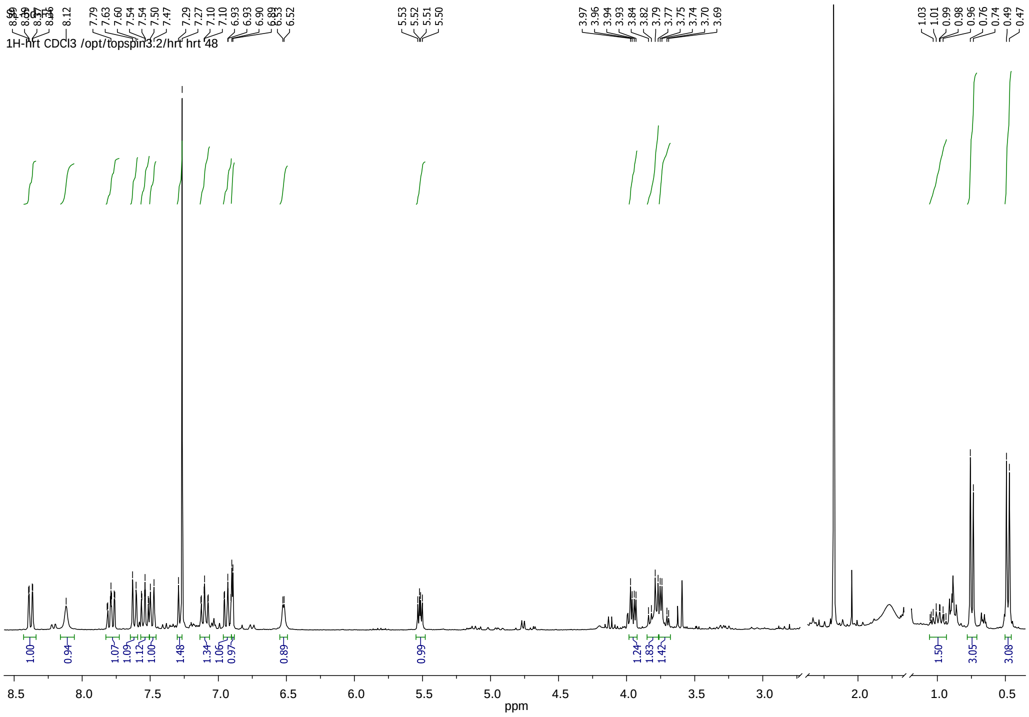

Figure S26. ^{1}H NMR spectrum of (1*R*,4*R*)-4-(1*H*-indol-3-ylmethyl)-1-isopropyl-2*H*-pyrazino[2,1-*b*]quinazolin-3,6-(1*H*, 4*H*)-dione (**4d**) ($CDCl_3$, 300, MHz).

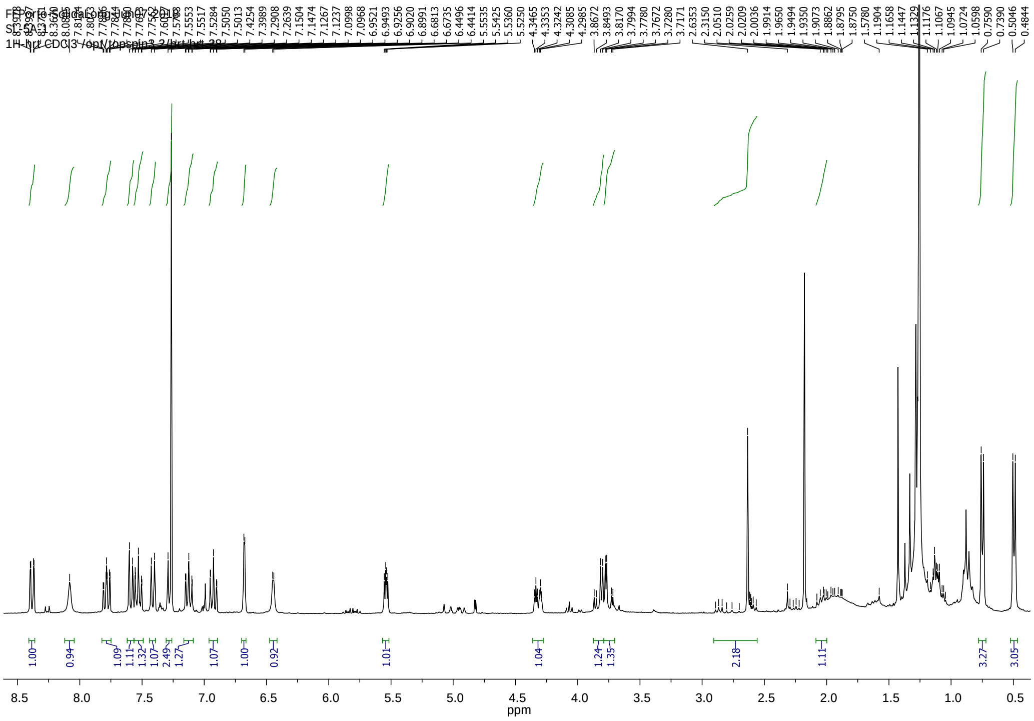

Figure S27. ^{1}H NMR spectrum of (1*S*,4*S*)-4-(1*H*-indol-3-ylmethyl)-1-isobutyl-2*H*-pyrazino[2,1-*b*]quinazolin-3,6-(1*H*, 4*H*)-dione (**5a**) ($CDCl_3$, 300, MHz).

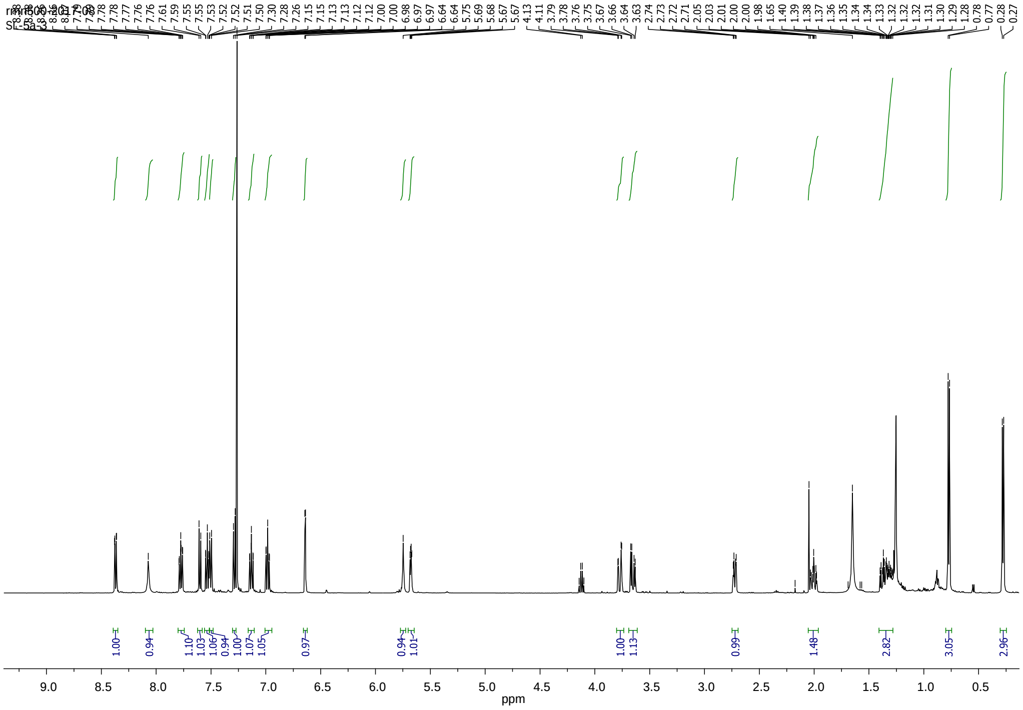

Figure S28. ^{1}H NMR spectrum of (1*R*,4*S*)-4-(1*H*-Indol-3-ylmethyl)-1-isobutyl-2*H*-pyrazino[2,1-*b*]quinazoline-3,6-(1*H*,4*H*)-dione (**5b**) ($CDCl_3$, 300, MHz).

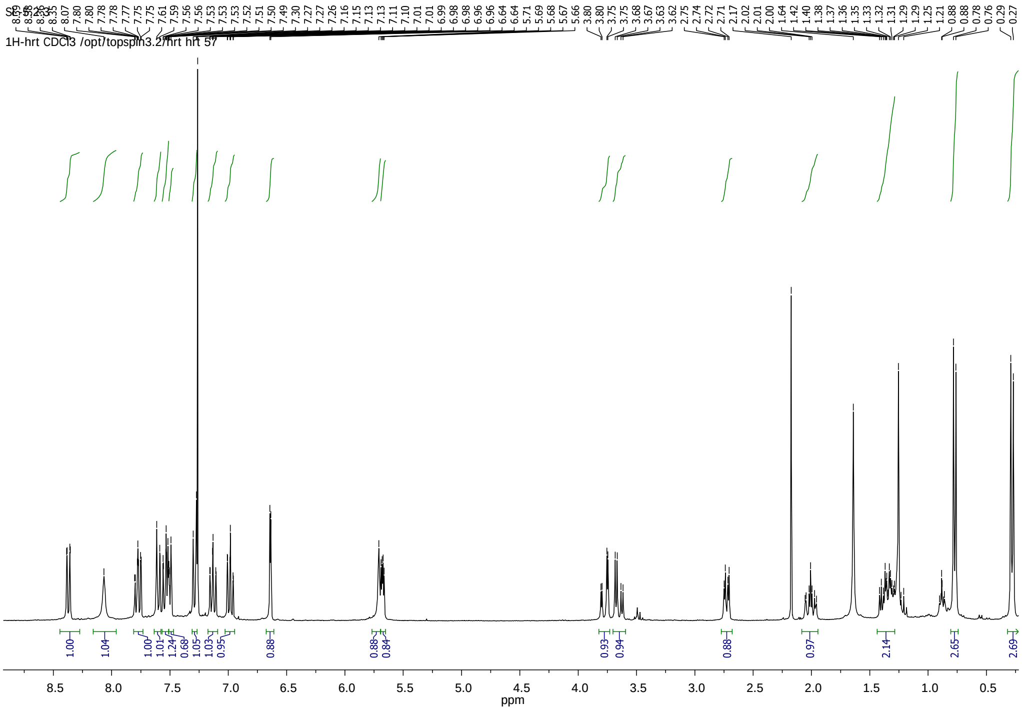

Figure S29. ^{1}H NMR spectrum of (1*S*,4*R*)-4-(1*H*-Indol-3-ylmethyl)-1-isobutyl-2*H*-pyrazino[2,1-*b*]quinazoline-3,6-(1*H*,4*H*)-dione (**5c**) ($CDCl_3$, 300, MHz).

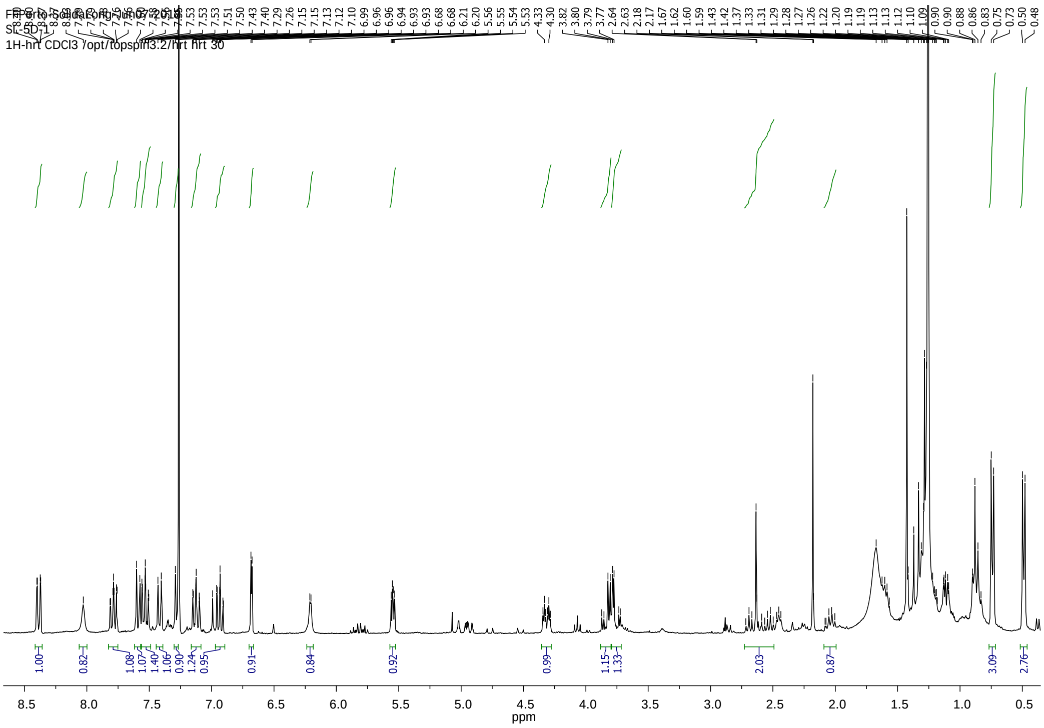

Figure S30. ^{1}H NMR spectrum of (1*R*,4*R*)-4-(1*H*-indol-3-ylmethyl)-1-isobutyl-2*H*-pyrazino[2,1-*b*]quinazolin-3,6-(1*H*, 4*H*)-dione (**5d**) ($CDCl_3$, 300, MHz).

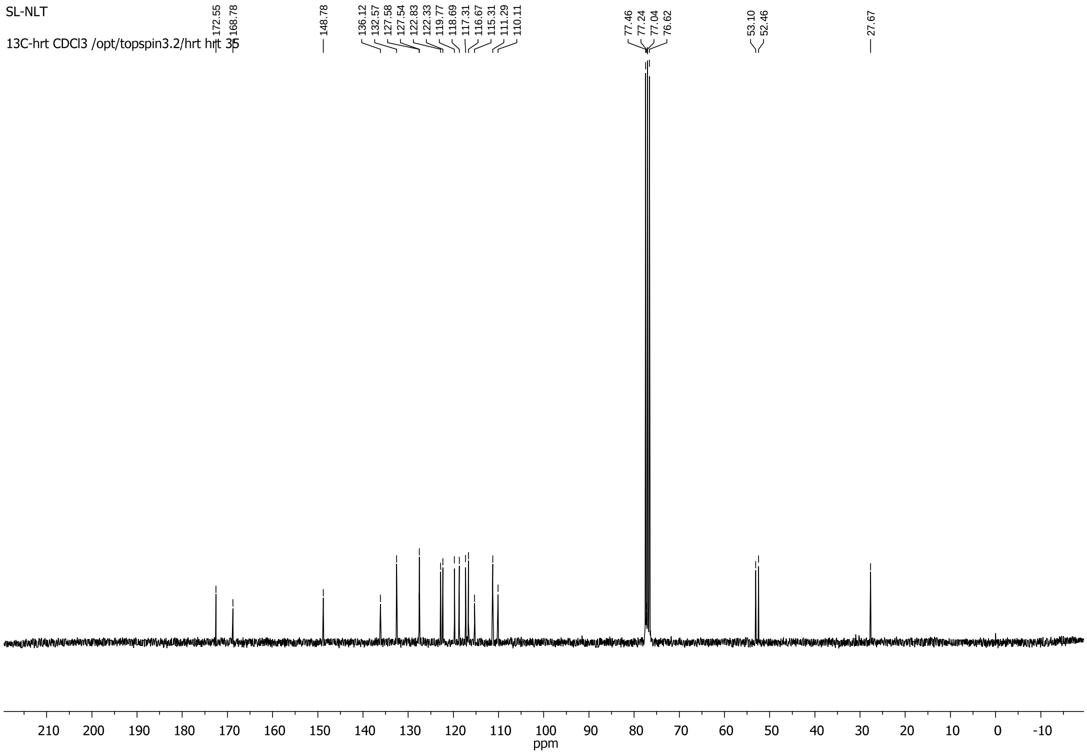

Figure S31. ^{13}C NMR spectrum of *N*-(2-aminobenzoyl)-L-tryptophan methyl ester **(iv-a)** ($CDCl_3$, 75, MHz).

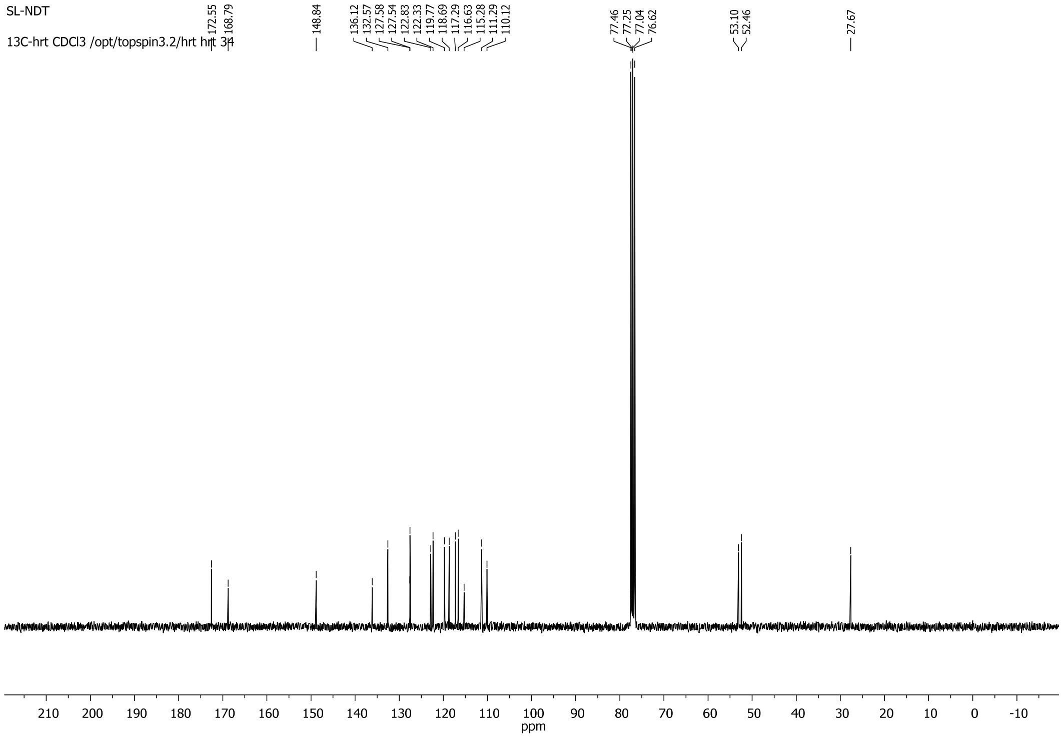

Figure S32. ^{13}C NMR spectrum of *N*-(2-aminobenzoyl)-D-tryptophan methyl ester **(iv-b)** (CDCl$_3$, 75, MHz).

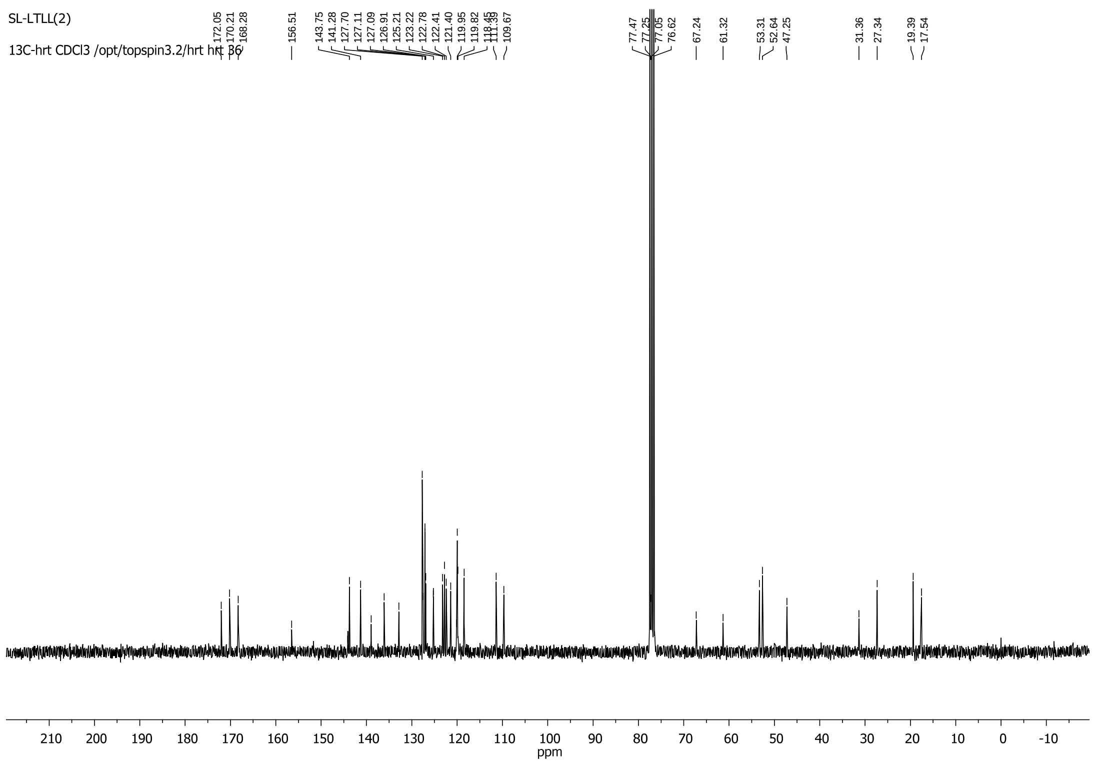

Figure S33. ^{13}C NMR spectrum of *N*-[9*H*-fluoren-9-ylmethoxy)carbonyl]-L-valinyl-2-aminobenzoyl-L-tryptophan methyl ester (**vi-a**) (CDCl$_3$, 75, MHz).

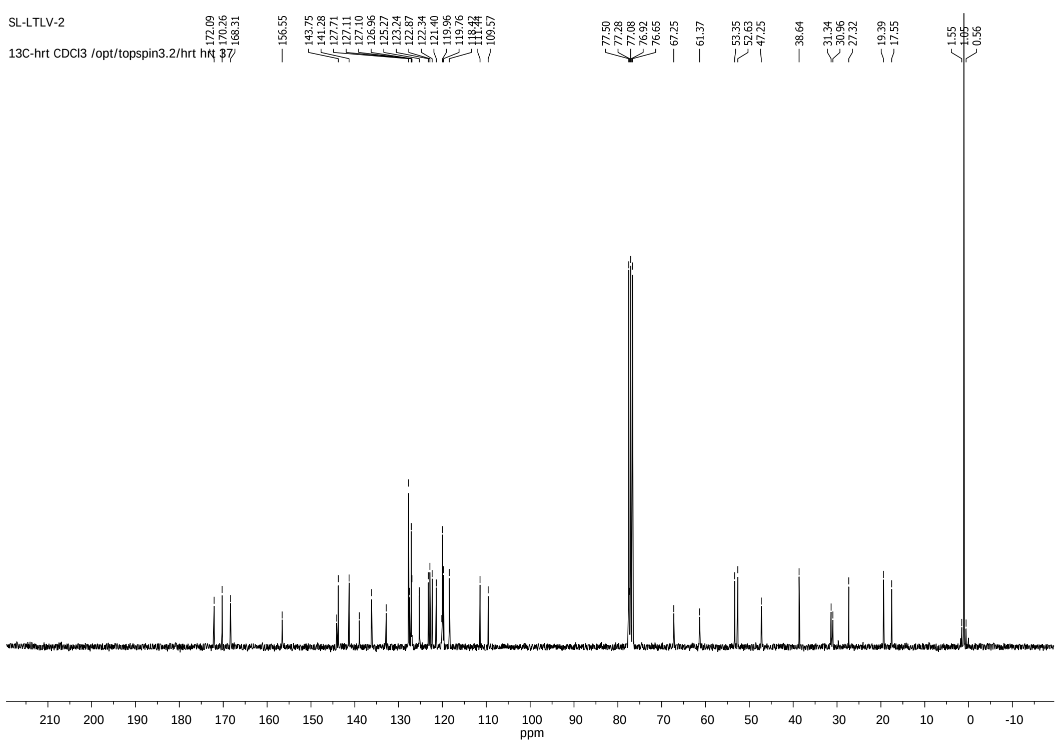

Figure S34. ^{13}C NMR spectrum of *N*-[9*H*-fluoren-9-ylmethoxy)carbonyl]-L-methylpentanyl-2*H*-aminobenzoyl-L- tryptophan methyl ester (**vi-b**) ($CDCl_3$, 75, MHz).

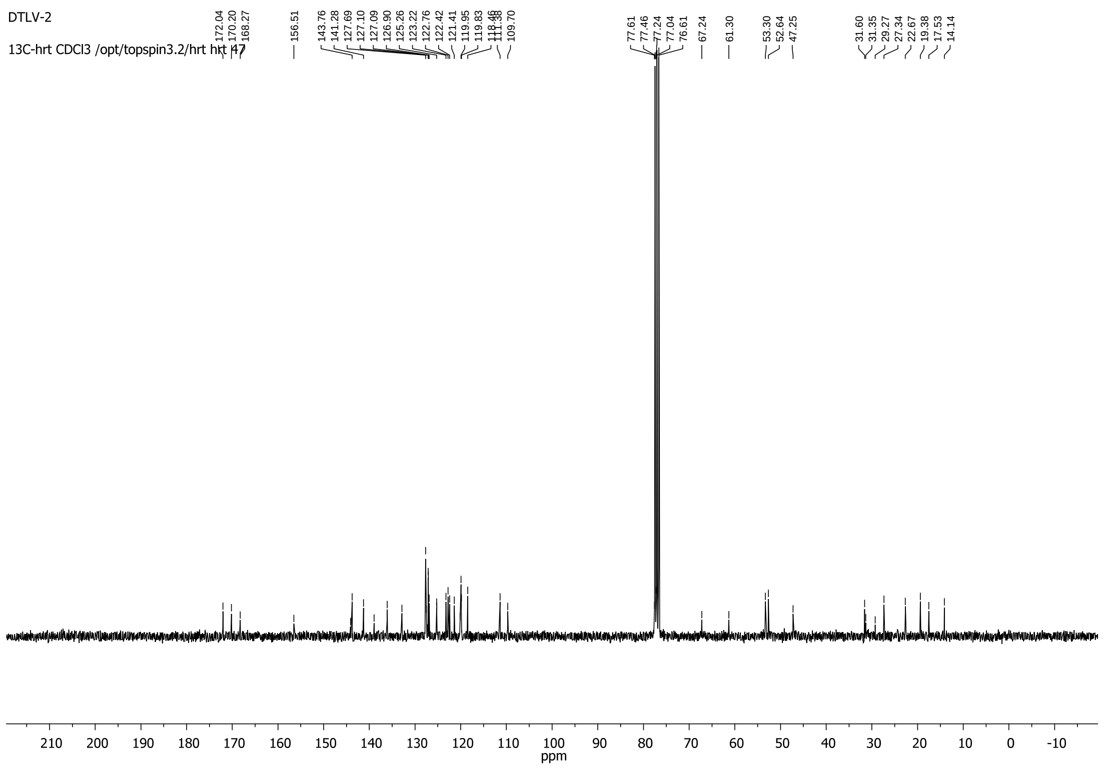

Figure S35. ^{13}C NMR spectrum of *N*-[9*H*-fluoren-9-ylmethoxy)carbonyl]-D-valinyl-2-aminobenzoyl-D-tryptophan methyl ester (**vi-c**) (CDCl$_3$, 75, MHz).

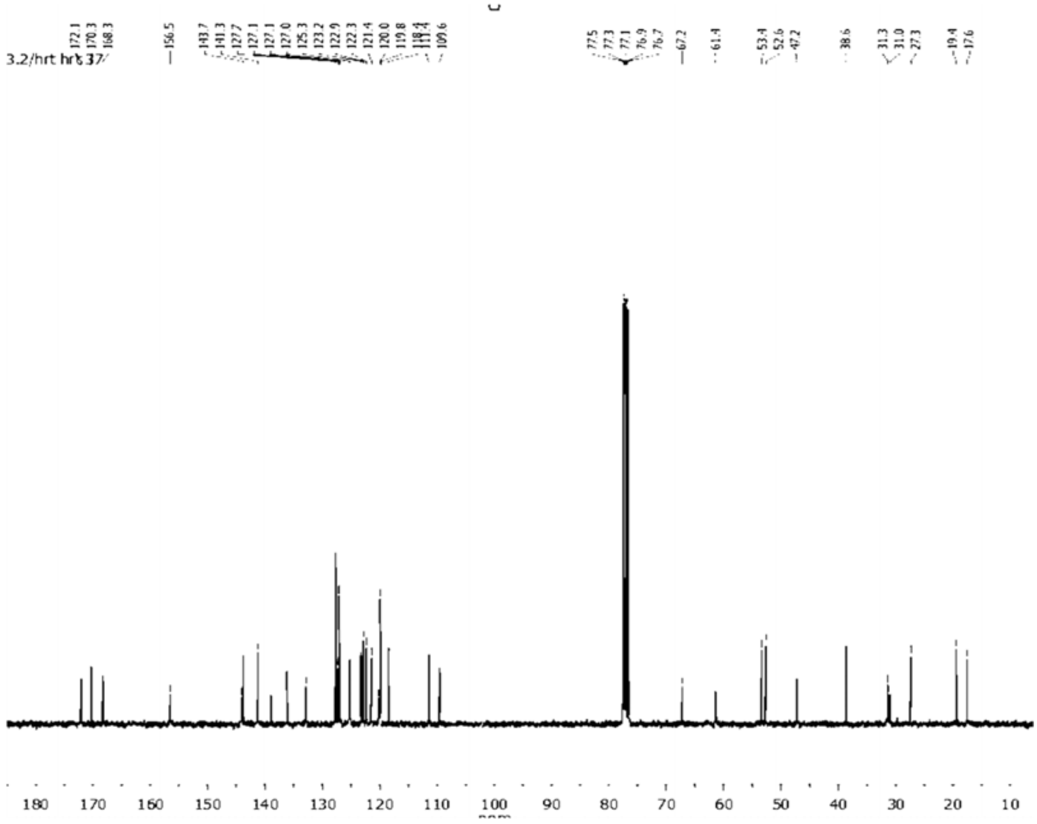

Figure S36. ^{13}C NMR spectrum of *N*-[9*H*-fluoren-9-ylmethoxy)carbonyl]-D-methylpentyl-2-aminobenzoyl-D- tryptophan methyl ester (**vi-d**) (CDCl$_3$, 75, MHz).

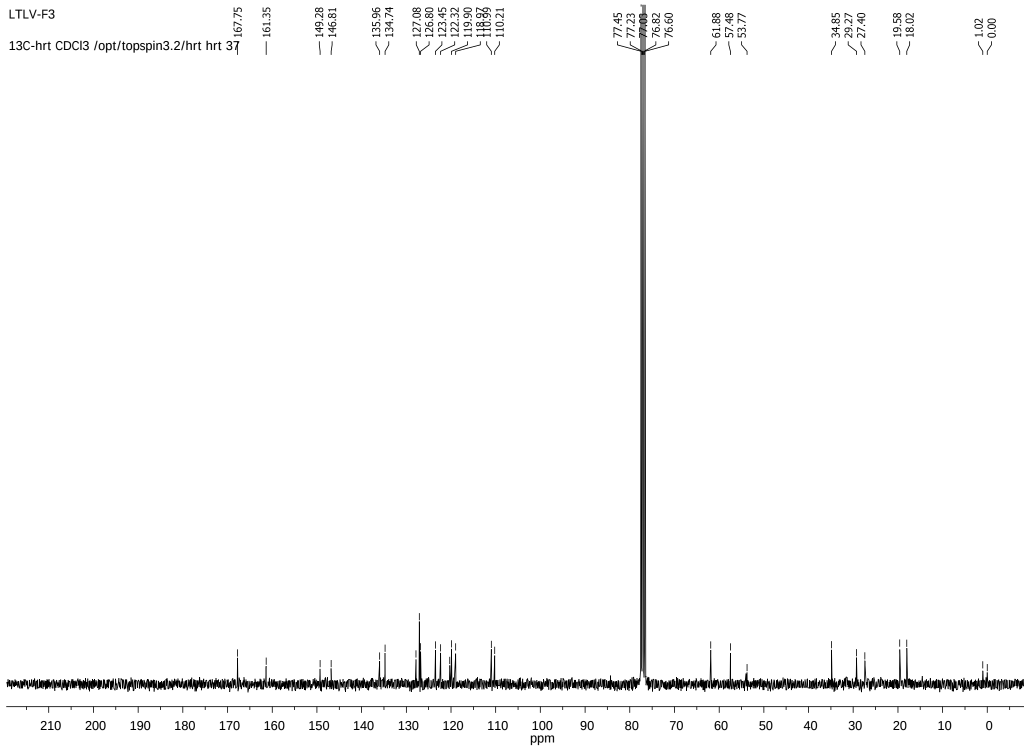

Figure S37. ^{13}C NMR spectrum of (1*S*,4*S*)-4-(1*H*-indol-3-ylmethyl)-1-isopropyl-2*H*-pyrazino[2,1-*b*]quinazolin-3,6-(1*H*, 4*H*)-dione (**4a**) ($CDCl_3$, 75, MHz).

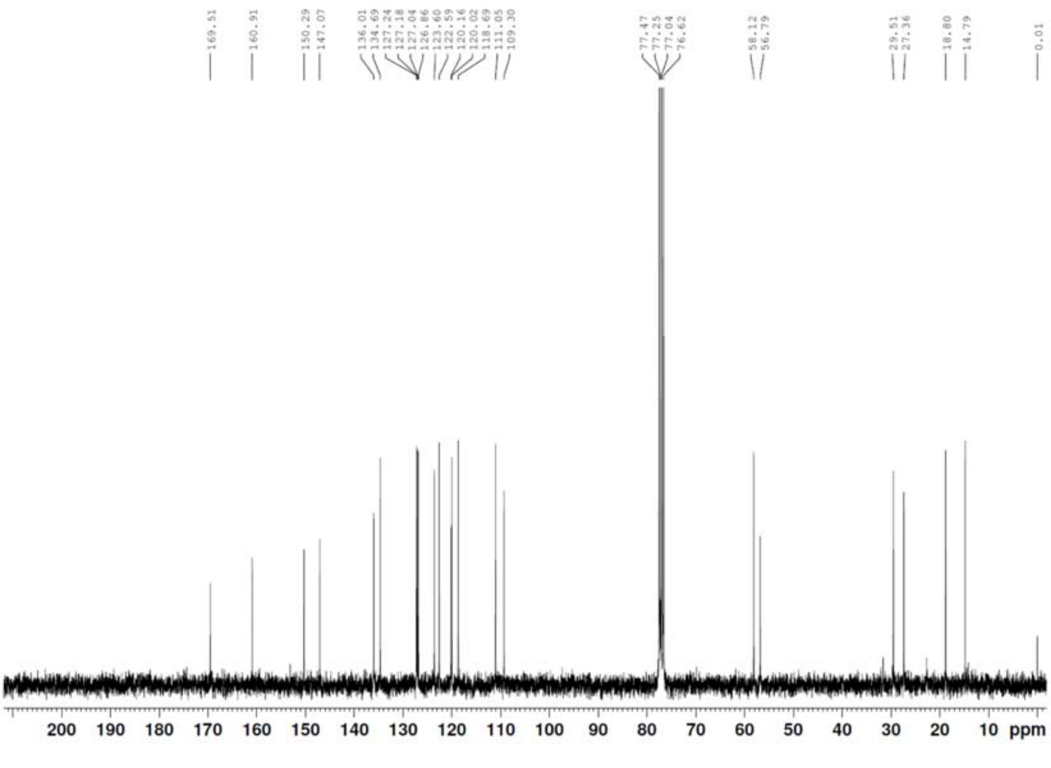

Figure S38. ^{13}C NMR spectrum of (1*R*,4*S*)-4-(1*H*-Indol-3-ylmethyl)-1-isopropyl-2*H*-pyrazino[2,1-*b*]quinazoline-3,6-(1*H*,4*H*)-dione (**4b**) ($CDCl_3$, 75, MHz).

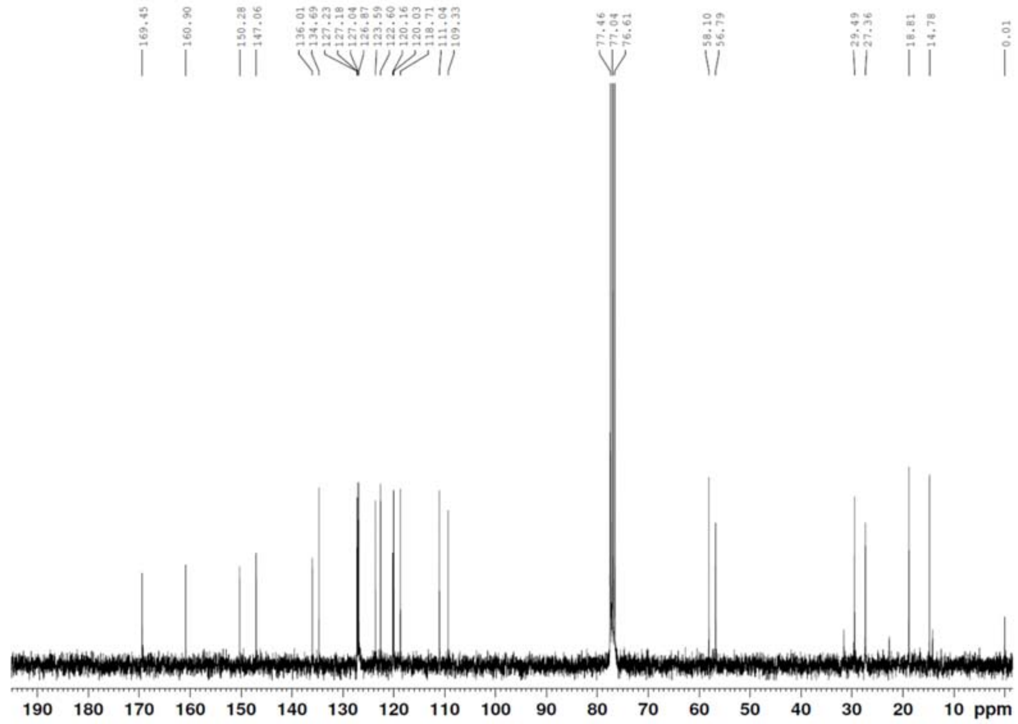

Figure S39. ^{13}C NMR spectrum of (1*S*,4*R*)-4-(1*H*-Indol-3-ylmethyl)-1-isopropyl-2*H*-pyrazino[2,1-*b*]quinazoline-3,6-(1*H*,4*H*)-dione (**4c**) ($CDCl_3$, 75, MHz).

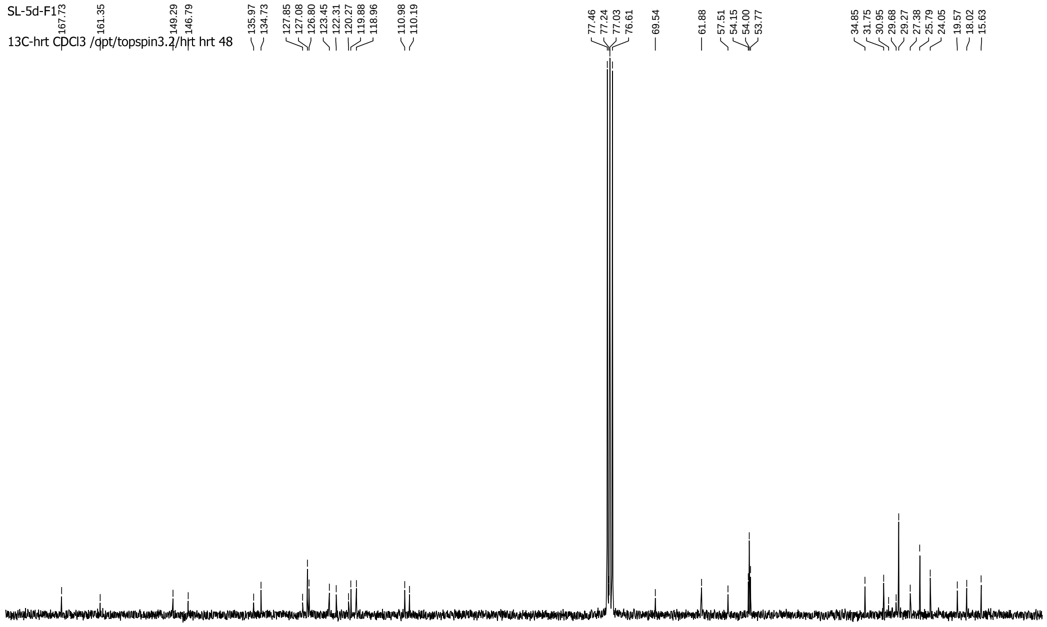

Figure S40. ^{13}C NMR spectrum of (1*R*,4*R*)-4-(1*H*-indol-3-ylmethyl)-1-isopropyl-2*H*-pyrazino[2,1-*b*]quinazolin-3,6-(1*H*, 4*H*)-dione (**4d**) ($CDCl_3$, 75, MHz).

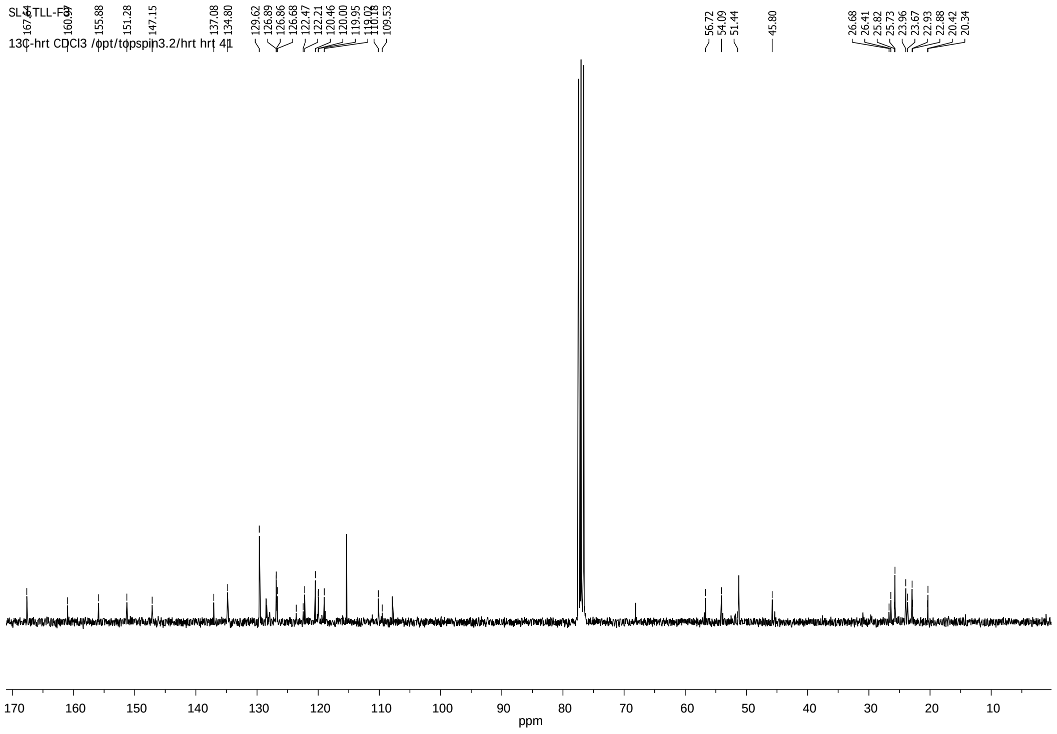

Figure S41. ^{13}C NMR spectrum of (1*S*,4*S*)-4-(1*H*-indol-3-ylmethyl)-1-isobutyl-2*H*-pyrazino[2,1-*b*]quinazolin-3,6-(1*H*, 4*H*)-dione (**5a**) (CDCl$_3$, 75, MHz).

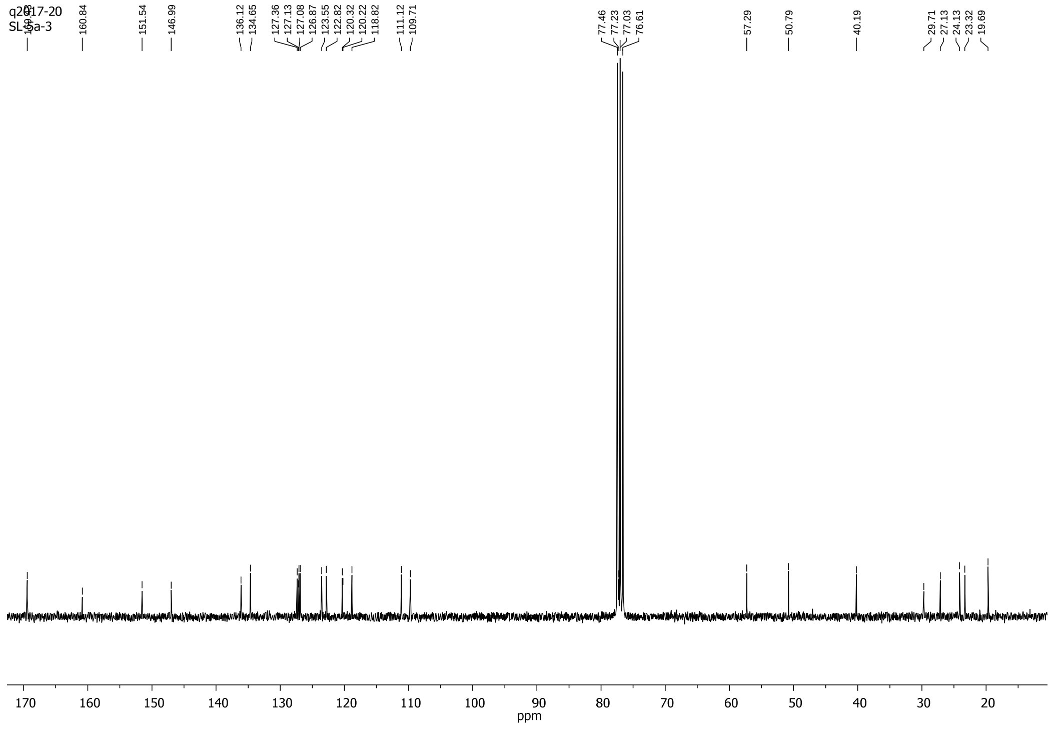

Figure S42. ^{13}C NMR spectrum of (1*R*,4*S*)-4-(1*H*-Indol-3-ylmethyl)-1-isobutyl-2*H*-pyrazino[2,1-*b*]quinazoline-3,6-(1*H*,4*H*)-dione (**5b**) (CDCl$_3$, 75, MHz)

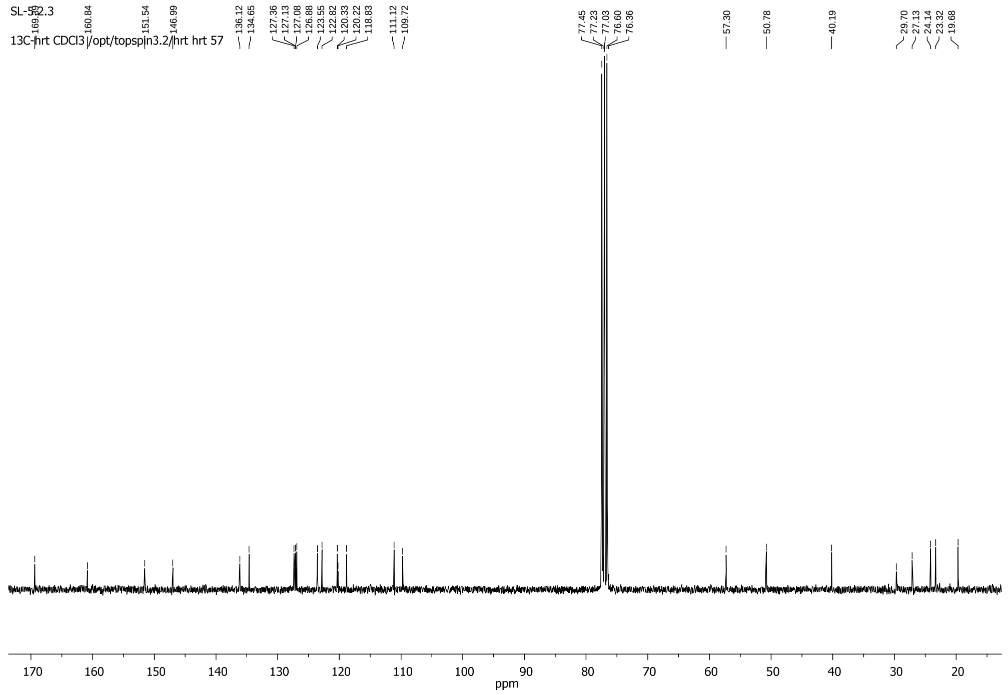

Figure S43. ^{1}C NMR spectrum of (1*S*,4*R*)-4-(1*H*-Indol-3-ylmethyl)-1-isobutyl-2*H*-pyrazino[2,1-*b*]quinazoline-3,6-(1*H*,4*H*)-dione (**5c**) ($CDCl_3$, 75, MHz).

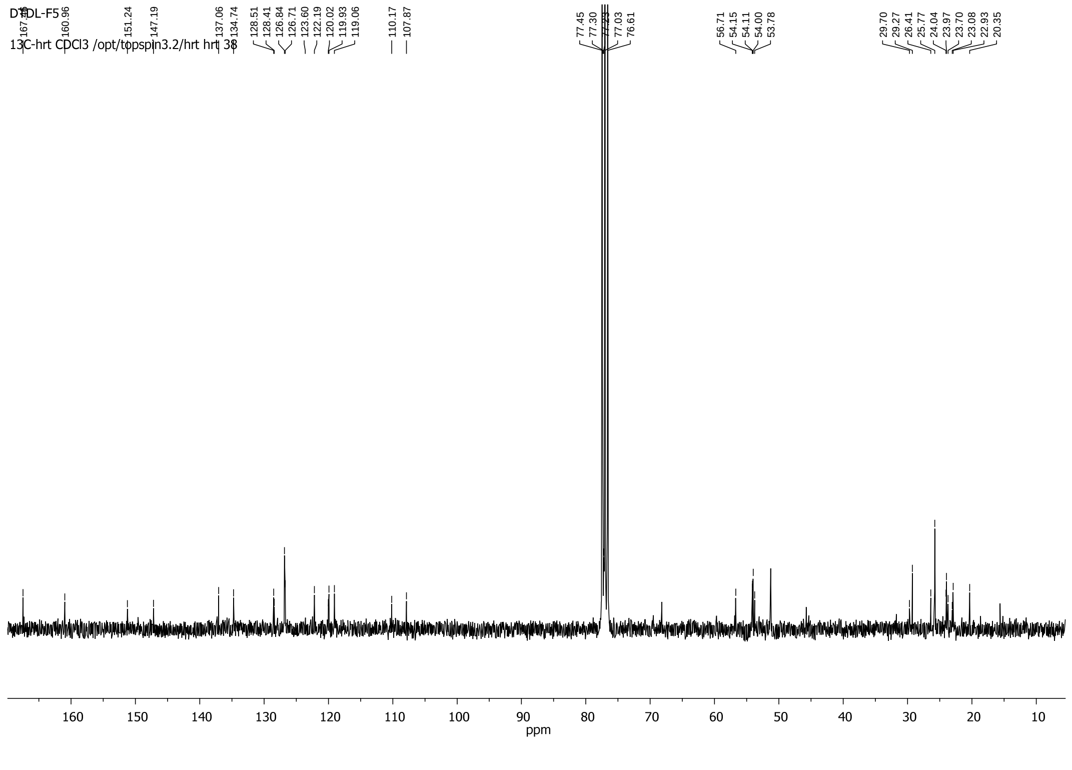

Figure S44. ^{13}C NMR spectrum of (1*R*,4*R*)-4-(1*H*-indol-3-ylmethyl)-1-isobutyl-2*H*-pyrazino[2,1-*b*]quinazolin-3,6-(1*H*, 4*H*)-dione (**5d**) ($CDCl_3$, 75, MHz).

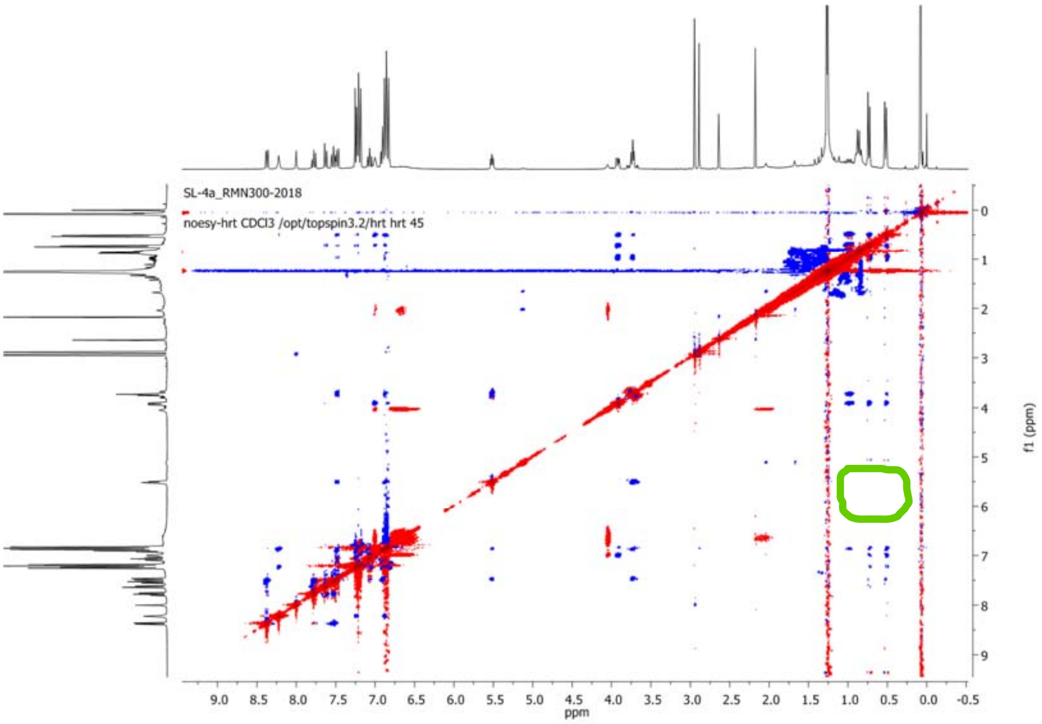

Figure S45. NOESY spectrum of (1*S*,4*R*)-4-(1*H*-Indol-3-ylmethyl)-1-isopropyl-2*H*-pyrazino[2,1-*b*]quinazoline-3,6-(1*H*,4*H*)-dione (**4c**) ($CDCl_3$, 300, MHz). The green circle represents no correlation of proton of C-1′ methyl group and H-4.

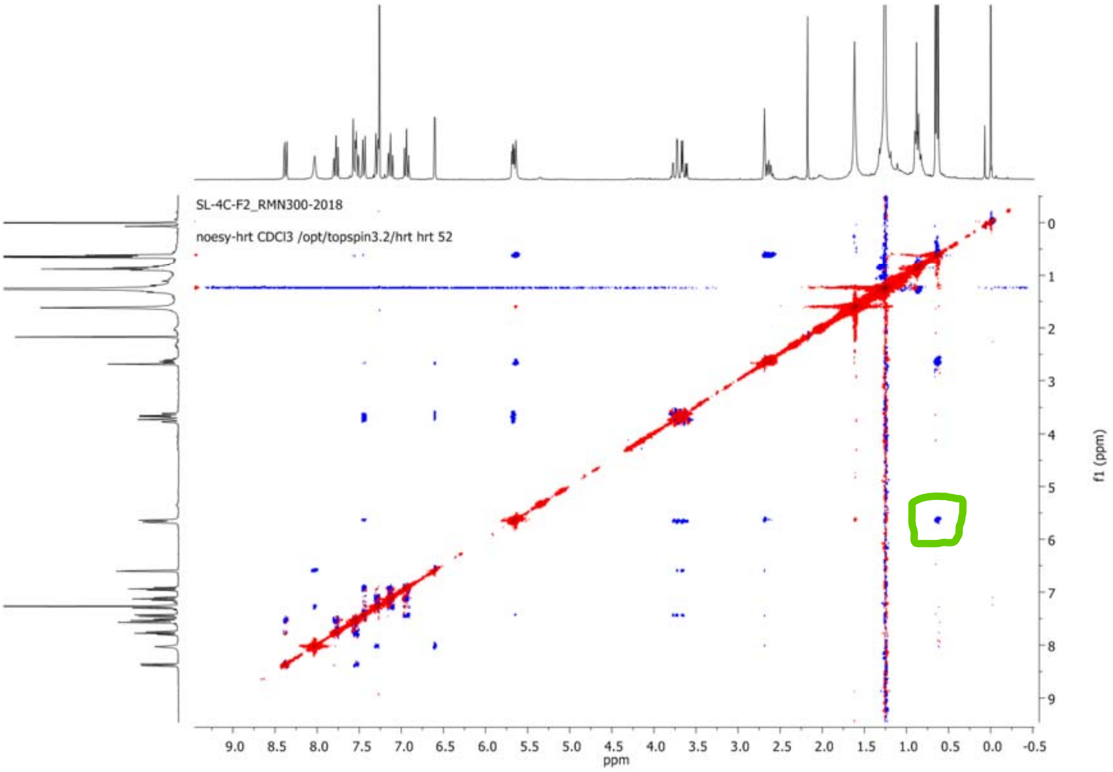

Figure S46. NOESY spectrum of (1*S*,4*S*)-4-(1*H*-Indol-3-ylmethyl)-1-isopropyl-2*H*-pyrazino[2,1-*b*]quinazoline-3,6-(1*H*,4*H*)-dione (**4a**) ($CDCl_3$, 300, MHz). The green circle represents the correlation of proton of C-1′ methyl group and H-4
